# Identifying quantitatively differential chromosomal compartmentalization changes and their biological significance from Hi-C data using DARIC

**DOI:** 10.1186/s12864-023-09675-w

**Published:** 2023-10-13

**Authors:** Yan Kai, Nan Liu, Stuart H. Orkin, Guo-Cheng Yuan

**Affiliations:** 1 Cancer and Blood Disorders Center, Boston Children’s Hospital and Dana-Farber Cancer Institute, Harvard Medical School, Boston, MA 02115 USA; 2 Bone Marrow Transplantation Center of the First Affiliated Hospital, Zhejiang University School of Medicine, Hangzhou, 310003 China; 3 Liangzhu Laboratory, Zhejiang University Medical Center, Hangzhou, 311121 China; 4 Howards Hughes Medical Institute, Boston, MA 02115 USA; 5 Department of Genetics and Genomic Sciences, Icahn School of Medicine at Mount Sinai, Charles Bronfman Institute for Precision Medicine, New York, NY 10029 USA

**Keywords:** Compartment, Compartmentalization, Hi-C, 3D genome, Gene regulation, Nuclear positioning

## Abstract

**Background:**

Chromosomal compartmentalization plays a critical role in maintaining proper transcriptional programs in cell differentiation and oncogenesis. However, currently the prevalent method for comparative analysis of compartmentalization landscapes between different cell types is limited to the qualitative switched compartments.

**Results:**

To identify genomic regions with quantitatively differential compartmentalization changes from genome-wide chromatin conformation data like Hi-C, we developed a computational framework named DARIC. DARIC includes three modules: compartmentalization quantification, normalization, and differential analysis. Comparing DARIC with the conventional compartment switching analysis reveals substantial regions characterized by quantitatively significant compartmentalization changes without switching. These changes are accompanied by changes in gene expression, chromatin accessibility, H3K27ac intensity, as well as the interactions with nuclear lamina proteins and nuclear positioning, highlighting the functional importance of such quantitative changes in gene regulation. We applied DARIC to dissect the quantitative compartmentalization changes during human cardiomyocyte differentiation and identified two distinct mechanisms for gene activation based on the association with compartmentalization changes. Using the quantitative compartmentalization measurement module from DARIC, we further dissected the compartment variability landscape in the human genome by analyzing a compendium of 32 Hi-C datasets from 4DN. We discovered an interesting correlation between compartmentalization variability and sub-compartments.

**Conclusions:**

DARIC is a useful tool for analyzing quantitative compartmentalization changes and mining novel biological insights from increasing Hi-C data. Our results demonstrate the functional significance of quantitative compartmentalization changes in gene regulation, and provide new insights into the relationship between compartmentalization variability and sub-compartments in the human genome.

**Supplementary Information:**

The online version contains supplementary material available at 10.1186/s12864-023-09675-w.

## Introduction

Mammalian genomes are structurally organized within the three-dimensional space of nucleus [1–3]. Spatial compartments are formed to facilitate functional partitioning of transcriptional activities [2, 4, 5]. Developments in technologies mapping genome-wide chromatin conformation, such as Hi-C and its derivatives [5–8], have greatly expanded our knowledge of these spatial compartments. There are two main types of compartments, transcriptionally permissive compartment A and repressive compartment B [6]. The two types of compartments are strongly correlated with gene density, distribution of CpG islands, histone modification marks, and replication timing [6, 9]. Structurally, the two types of compartments preferentially occupy different spaces within the nucleus [10, 11]. For example, experiments [12–14] mapping nuclear lamina-associated chromosomal domains reveal that compartment B preferentially occupies the periphery regions in the nucleus. In addition, recent advances in genome positioning mapping [15–17] further reveal a close link between A/B compartment distribution and the positioning in the lamina-to-speckle axis. Mechanistically, emerging studies [18, 19] show that liquid–liquid phase separation caused by interactions among high concentrations of multivalent proteins and other biological molecules like RNA plays a crucial role in compartment formation.

There are extensive changes in genome compartmentation patterns between different cell types or cellular conditions, and these changes play a crucial role in the activation and repression of genes [20–23]. For example, Dixon et al. [20] studied compartment reorganization in human embryonic stem cells and four derived lineages, and reported 36% of genome switch compartments in at least one of the lineages. Johnstone et al. [24] comprehensively examined the compartment differences between colon tumors and normal samples and found that the normal compartment structures are profoundly compromised in tumors. Further integrated analysis [24] with functional genomics data reveals that those compartment changes are accompanied by profound changes in DNA methylation and chromatin states, highlighting the functional consequences of compartment disorganization. Due to critical roles of compartments in development and diseases, it is of keen interest to identify the differential compartments between cell types or cells in different conditions.

Currently, the prevalent approach for differential compartment analysis is to identify switched compartments, i.e. the genomic regions that flip from compartment A to B, or the opposite, between two cell types. Specifically, this analysis involves two steps [5]. First, compartment types are identified in each cell type by performing the Principal Components Analysis (PCA) of the chromatin interaction matrix after distance-normalization and transformation into a correlation matrix at a selected resolution. The genomic bins can be divided into two types, positive or negative, by their values of the first principal component (PC1 value). The sign of the PC1 values (i.e. positive or negative) represents the two types of compartments, and gene density is then used to further determine that the sign corresponding to higher gene density represents compartment A. After compartments are defined in each cell type, the signs of each genomic region are compared and the genomic regions that are characterized by flipped compartments are defined as the differential ones. This compartment switching analysis method is limited in many ways. First, the PCA is performed for each sample separately, thus making the PC1 values not directly comparable between samples. Second, PC1 values lack a clear biological meaning in compartmentalization. Third, this approach lacks the flexibility to be expanded to compartmental differences across multiple cell types. Furthermore, this analysis by nature is qualitative and ignores the genomic regions that are characterized by quantitatively differential compartment domains. Therefore, a quantitative approach with interpretable biological meaning is needed.

Here we bridged these gaps by developing a computational framework, named DARIC (Differential Analysis of genomic Regions’ Interactions with Compartments), to find genomic regions with quantitatively differential compartmentalization changes from genome-wide chromatin conformation data like Hi-C. DARIC is a comprehensive framework including compartmentalization quantification, normalization, and differential analysis. We designed a metric, named Preferential Interaction Score (PIS), to quantify the compartment type preference and strength. Furthermore, we showed that DARIC is robust to technical variations in Hi-C, such as choices of different restriction enzymes and sequencing depth. Comparison between DARIC with the conventional compartment switching analysis reveals substantial regions characterized by quantitatively significant compartmentalization changes without switching. More importantly, integrative analysis with functional genomics data demonstrates that such quantitatively differential regions are associated with concordant changes in gene expression, chromatin accessibility, H3K27ac intensity, as well as the interaction with nuclear lamina proteins and radial positioning within the nucleus, highlighting the functional importance of the regions with quantitatively differential compartment strength. To demonstrate the utility of DARIC, we first applied DARIC to public time-course Hi-C datasets delineating the differentiation of human cardiomyocytes from embryonic stem cells. Interestingly, we found that activated genes in cardiomyocytes can be divided into two groups that have distinct characteristics, such as local abundance of regulatory elements and function specificity, by whether or not associated with significant compartmentalization changes. We also applied DARIC to a compendium of Hi-C data in the 4D Nucleosome consortium and performed an unbiased evaluation of compartmentalization variability analysis in the human genome. We found a surprising correspondence between different types of sub-compartments and PIS variability. In summary, DARIC provides a unique tool for finding quantitative compartmental differences between cell types that are otherwise impossible from the conventional switching analysis.

## Materials and methods

### Implementation of DARIC

#### Preferential Interaction Score calculation

It takes two types of information for PIS calculation: A/B compartments and the distance-normalized chromatin interaction files. A/B compartments can be obtained from any Hi-C compartment identification algorithms, such as the PCA analysis from Homer [25] or eigenvector analysis from Juicer [26]. For chromatin interaction files, we used the obs/exp normalized interactions to eliminate the bias caused by genomic bias. These interaction files are obtained from the “.hic” files by using the “java -jar juicer_tools.jar dump oe KR” command from Juicer. PIS was then calculated for each chromosome individually. Specifically, for each genomic bin, the average interactions with compartment A and B bins in the same chromosome were calculated and the log2-transformed ratio of the average interactions was calculated as the PIS. To avoid biases, the gap regions in the reference genome assemblies, to which sequencing reads cannot be mapped, are excluded for PIS calculation.

#### Smoothing

After obtaining the raw PIS track, we performed 1-dimensional gaussian smoothing using the “gaussian_filter1d” function from the scipy package [27].

#### Normalization

We borrowed the idea from MA-norm [28], a method originally developed from ChIP-seq data normalization, to normalize PIS tracks from two Hi-C samples. Similar to ChIP-seq data, we make two assumptions with the usage of MA-norm: (a) most genomic regions do not have significant changes in compartmentalization. This assumption is valid as suggested by chromatin conformation data across many tissue- and cell- types. (b) the scaling relationship of PIS between two Hi-C samples is reflected in observed PIS differences in most genomic regions, which can be extrapolated to all genomic regions. The (M, A) value of each genomic bin is defined as $\left(PIS1-PIS2, \frac{PIS1+PIS2}{2}\right).$ The scaling relationship of PIS between two Hi-C samples is derived from background regions, which are defined as genomic regions whose residual PIS values (i.e. the $M$ values) are ranked in 15% to 85% percentile. Similar to the implementation of the original MA-norm package, we used robust regression to the (M, A) values of the background regions to derive a linear relationship [28]. The linear relationship is then extrapolated to all genomic regions by making the A axis overlap with the derived linear model, and the new M value from the derived linear model is considered as the normalized M value. The normalized PIS track is then obtained by adding the normalized M value to the PIS values of the reference sample.

#### Identification of differential domains

To identify the quantitatively differential domains in compartmentalization, we applied a Hidden Markov Model (HMM, https://hmmlearn.readthedocs.io/en/latest/) to segment the genome into four states based on the residual PIS track. We chose four as the state number to make it correspond to the four states in the conventional compartment switching analysis.

#### Statistical analysis

If biological replicates are available, DARIC can test the statistical significance of the PIS differences between two cell types based on PIS differences between biological replicates in each cell type. This method was previously used in the statistical significance estimation of TSA-seq signal differences [17]. Essentially, the PIS differences between biological replicates are used to build an empirical null distribution, and the statistical significance of differences between the two cell types is tested. PIS differences between two cell types are defined as the residuals of the mean PIS of the replicates, i.e.

$$\Delta PIS= \frac{{PIS}_{(Cell1, Rep1)}+{PIS}_{(Cell1, Rep2)}}{2}-\frac{{PIS}_{(Cell2, Rep1)}+{PIS}_{(Cell2, Rep2)}}{2}$$

where $Cell1$ and $Cell2$ are two cell types.

To construct the null distribution of PIS differences, we first used the average PIS differences between biological replicates in all possible orderings to build a vector $\varnothing$. $\varnothing$ has a length of $4N$, where $N$ is the number of genomic bins.

$$\varnothing =\frac{1}{2}(\left({PIS}_{Cell1, Rep1}-{PIS}_{Cell1, Rep2}\right)+\left({PIS}_{Cell2, Rep1}-{PIS}_{Cell2, Rep2}\right), \left({PIS}_{Cell1, Rep2}-{PIS}_{Cell1,Rep1}\right)+\left({PIS}_{Cell2,Rep1}-{PIS}_{Cell2, Rep2}\right), \left({PIS}_{Cell1,Rep1}-{PIS}_{Cell1,Rep2}\right)+\left({PIS}_{Cell2, Rep2}-{PIS}_{Cell2,Rep1}\right), \left({PIS}_{Cell1,Rep2}-{PIS}_{Cell1,Rep1}\right)+\left({PIS}_{Cell2,Rep2}-{PIS}_{Cell2,Rep1}\right))$$

$\varnothing$ can be taken as a Gaussian distribution approximately, which always has a mean of 0 due to the symmetry in $\varnothing$ construction.

For each genomic bin, we then estimated the one-sided p-value for residual PIS between two cell types in the null distribution $\varnothing$. We then performed $-{log}_{10}pvalue$ transformation to obtain a significance score and used this score for display in the genome browser. To avoid extreme values in significance scores, p-values more significant than 1e-20 were considered as 1e-20. Domains characterized with significant PIS changes, i.e. “Strong ± ” domains from the previous step, with an average significance score higher than 2 were deemed as significant.

### Hi-C data processing in H1ESC and K562

Hi-C data for H1ESC and K562, each with two biological replicates, were downloaded from 4DN [6, 8, 13] (See accession numbers in Table S1). Specifically, we downloaded the contact read pairs processed by 4DN. With the read pairs, we constructed contact maps in “.hic” format with obs-exp and KR normalization using Juicer [26]. A/B compartments were then identified at 50 kb resolution by using the “runHiCpca.pl” script from Homer [25].

### Comparison of functional genomics data between H1ESC and K562

All used functional genomics data in the H1ESC-K562 comparison, including RNA-seq [29], DNase-seq [29], H3K27ac ChIP-seq [29], Anti Son TSA-seq [17], and Lamin B1 DamID data [13], are summarized in Table S1. For RNA-seq data, the downloaded bam files were used to obtain the raw count table for each gene using the “featureCounts” command [30]. Differential expression analysis was performed using DESeq2 [31]. Genes with a *p*-value more significant than 1e-3 and log2FoldChange higher than 1 or lower than -1 were deemed as differentially expressed genes (DEGs). The enrichment of DEGs on the four types of domains (i.e. “Strong-”, “Weak-”, “Weak + ”, and “Strong + ”) were shown using the obs/exp fold enrichment. The expected number of DEGs in domain $i$ is based on the gene density of that domain, i.e.

$$N(expected, domain-i)=N\left(DEG\right)\times \frac{N(all-genes-in-domain-i)}{N(all-genes)}$$

Super-enhancers for H1ESC and K562 were downloaded from the dbSUPER database [32]. Cell-type-specific super-enhancers were defined as those showing no overlap (1 bp) with those in the other cell type. The enrichment analysis of super-enhancers in the four types of compartmentalization domains was performed with the same method used in DEG enrichment.

For TSA-seq and lamin B1 DamID data, the processed and normalized files in bigwig format were downloaded from 4DN. The signals for each genomic bin at 50 kb were extracted from bigwig files and comparisons were made between the four types of domains.

### Effects of restriction enzymes and sequencing depth

To study the effects of restriction enzymes on PIS, we downloaded three Hi-C datasets from 4DN, which are generated with three restriction enzymes for GM12878 cells: HindIII (4DNFII4JC7KV), DpnII (4DNFIDDMNL9R), MboI (4DNFIUOVQH68). Processed contact read pairs were downloaded and down-sized to the same sequencing depth (200 million read pairs). Subsequently, the same steps as described for H1ESC and K562 cells were taken, and the resulting PIS tracks were compared.

To study the effects of sequencing depth on PIS, we downloaded a deeply sequenced Hi-C dataset for H1ESC cells from 4DN (4DNFITU7K8VQ). There are more than 2 billion contact read pairs in the whole dataset. We down-sized this large dataset into eight different depths by random sampling: 150 m, 300 m, 450 m, 600 m, 900 m, 1200 m, 1500 m, and 2000 m, and prepared the PIS tracks for each sample as described above.

### Applying DARIC to time course Hi-C data during cardiomyocyte differentiation

Data used for delineating cardiomyocyte differentiation, including Hi-C, RNA-seq and H3K27ac ChIP-seq, were downloaded from GEO with accession number GSE116862 [33]. There are two biological replicates for Hi-C data at each time point. PIS tracks were prepared for each Hi-C sample as described above. For normalization, PIS at Day00 was used as the reference and PIS tracks at other time points were normalized with respect to Day00. After normalization, the averaged PIS of two replicates is used as the final value at each time point and shown in the genome browser. Residual PIS tracks were obtained for neighboring time points. DARIC was then applied to the four residual PIS tracks simultaneously to identify the genomic regions with significant compartmentalization changes. We applied DARIC to all the comparisons simultaneously to ensure that one unified HMM model was trained, thus the differential compartments from each comparison are based on the same model.

To examine the differences between activated genes associated with compartmentalization changes and those that do not, we performed differential gene expression analysis between ESCs (Day00) and primitive cardiomyocytes (Day15) using DESeq2 [31]. Cardiomyocyte-specific genes were defined as those with a p-value more significant than 1e-3 and log2Foldchange higher than 1. In total, 1948 cardiomyocyte-specific genes were identified. Genes whose TSS reside in any “Strong + ” domains in the time course were deemed as those associated with compartmentalization changes. ShinyGO [34] (v0.76) was used for Gene Ontology (Biological Process) enrichment analysis. Terms with less than 8 members were filtered out and the FDR cutoff was set to 0.05.

Loops used in this study were identified by applying FitHiC2 (v2.0.8) [35] to the day15 Hi-C data after merging replicates, with 5 kb as the resolution. Interactions with a q value smaller than 0.01 were identified as loops. Subsequently, loops whose anchor overlapped with promoter regions (TSS ± 2 kb) of cardiomyocyte-specific genes were considered relevant and were subject to loop length comparison.

### Compartmentalization variability analysis

Hi-C datasets, in the format of contact read pairs, for a compendium of cell types were downloaded from 4DN. Detailed information, including cell type, restriction enzyme, accession numbers, and labs that generated the data, are summarized in Table S2. In total, Hi-C data of 32 cell types were downloaded and examined. Visual inspection of the PC1 files in the genome browser filters out 13 cell types due to high-level noises and dis-continuity in the PC1 values. PIS tracks for the resulting 19 cell types were prepared as described above. To remove systematic biases caused by differences in protocols, all cell types’ PIS tracks were normalized with respect to H1ESC PIS. PIS tracks after normalization were shown in the genome browser and can be downloaded from https://github.com/ykai16/DARIC/tree/main/data.

To identify genomic regions displaying distinct levels of compartmentalization variability, we applied an HMM model to segment the genome into five states based on the standard deviation and mean of PIS across the 19 cell types. Enrichment analysis for functional genomic elements was performed using “annotatePeaks.pl” function from HOMER [25]. For the enrichment analysis of tissue-specific genes, we downloaded the gene expression specificity (Tau index) table calculated from the GTEx data from Palmer et.al [36]. For each tissue, a gene with a Tau index higher than 0.3 was deemed as specific to that tissue. Then the log2(obs/exp) enrichment analysis was performed in the same approach above. The sub-compartments for all the interrogated cell types were downloaded from Xiong et al. [37].

## Results

### Introduction of the DARIC framework

**Fig. 1:**
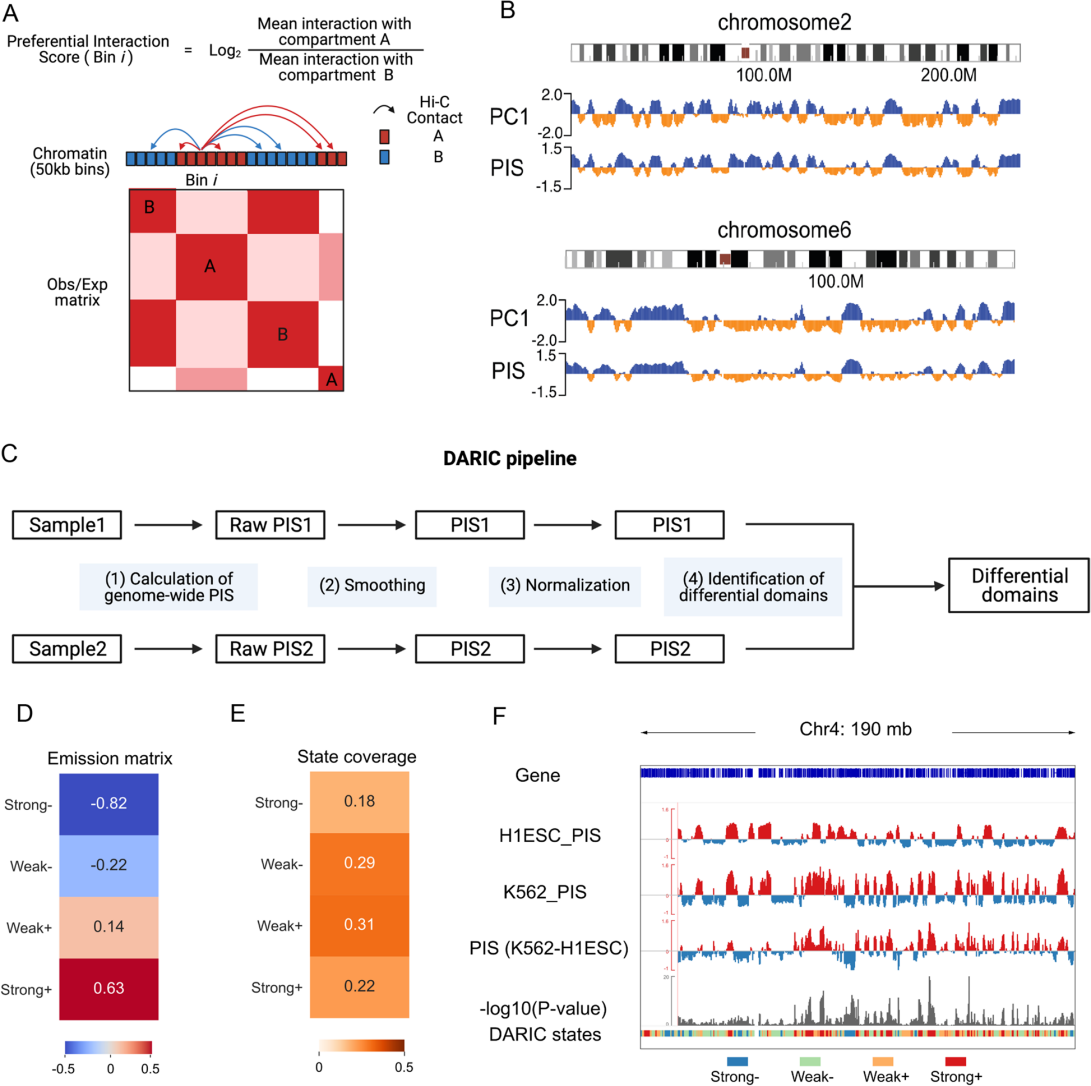
Introduction of the DARIC framework. **A** Schematic of the Preferential Interaction Score (PIS) definition. **B** Snapshots of chromosomes 2 and 6 showing the high correlation between PIS and PC1 values. **C** Flowchart of the DARIC pipeline for identifying genomic regions with significantly differential compartment changes. **D** Emission probability of the four-state Hidden Markov Model (HMM) model trained from the differential PIS between H1ESC and K562 cells. Values in the heatmap represent the mean value of PIS differences for each state. Strongly increased/decreased states are denoted as “Strong ± ”. Weakly increased/decreased states are denoted as “Weak ± ”. **E** Genomic coverage of the four states as shown in **D**. **F** Exemplar genome snapshot showing the PIS comparison of H1ESC and K562 and the output of DARIC, including statistical significance track (-log10P-value) and the segmentation of genome into four states

To quantify the compartmentalization strength for genomic regions, we first devised a metric named Preferential Interaction Score (PIS). PIS is defined as the log-transformed ratio of the average interactions with compartments A to B. Specifically, we first binned the genome at a selected size, usually at 100 kb or 50 kb, applied the PCA analysis, and obtained the compartment type information for each genomic bin. For each bin, the chromatin interactions with other A- and B-type bins in the same chromosome were calculated, and PIS was calculated subsequently (Fig. 1A). A positive/negative PIS indicates that the genomic region preferentially interacts with the active/repressive compartment, respectively. Moreover, a higher PIS represents stronger interaction with the active A compartment. Interestingly, we observed that PIS is highly correlated with the PC1 values (Fig. 1B, Figure S1A). Notwithstanding, we used PIS instead of PC1 values for further quantitatively differential analysis for mainly two reasons: (a) PIS has a clear biological meaning, enabling a straightforward interpretation of the quantitative differences; (b) Direct quantitative comparison of PC1 values for two Hi-C samples is not a legitimate approach, because PC1 values from two separate PCA analyses are not directly comparable.

DARIC includes the following four steps (Fig. 1C): (1) Calculation of the genome-wide PIS for the samples; (2) Smoothing of PIS in each sample to remove technical noises (see methods for details); (3) Normalization. Systematic differences, such as choices of restriction enzymes and heterogenous composition of cells in different cell cycle stages, are common confounding factors for Hi-C data comparison. To eliminate systematic biases, we borrowed a concept from MA-norm [28], a method originally developed for ChIP-seq data normalization, to normalize PIS tracks from two Hi-C samples. Similar to ChIP-seq data, we make two assumptions with the usage of MA-norm: (a) most genomic regions, by default defined as those ranked in the 15%-85% percentage in PIS residuals of the two cell-types, do not have significant changes in compartmentalization. This assumption is valid because chromatin conformation data across many tissue- and cell- types have suggested this. (b) the scaling relationship of PIS between two Hi-C samples is reflected in observed PIS differences in most genomic regions, which can be extrapolated to all genomic regions. Using the comparison between H1ESC and K562 as an illustrating example (Figure S1B and C), we showed that MA-norm can substantially eliminate the technical biases while still detecting biological differences between the two cell types; (4) Identifying differential domains and performing statistical analyses. Specifically, we used a Hidden Markov Model (HMM) to segment the genome into four states based on the residuals of two PIS tracks: Strongly decreased (“Strong-”), Weakly decreased (“Weak-”), Weakly increased (“Weak + ”) and Strongly increased (“Strong + ”), which are determined by the mean PIS changes of the four states (i.e. the emission matrix from the HMM model, Fig. 1D). We demonstrated that although the choice of a higher state number would reveal a finer resolution of compartmentalization changes, the regions showing strong PIS changes are largely overlapped across different models (Supplementary Fig. 1D-F). To facilitate the downstream analysis, we chose four as the state number to make it correspond to the four states in the conventional compartment switching analysis, i.e. the stable A or B compartments (“AA” or “BB”) and the switched compartments (“AB” and “BA”). Furthermore, if replicate data are available, DARIC enables the statistical significance analysis of PIS differences by using the variations within the replicates as an empirical background (see methods for details). In the comparison between H1ESC and K562, DARIC reveals that 40% of the genome (18% for decreased and 22% for increased PIS in K562) are identified with significant changes in compartmentalization (Fig. 1E). An example of the input and main outputs of DARIC are summarized in Fig. 1F.

### Quantitative PIS differences are associated with concordant changes in transcription and chromatin state

**Fig. 2:**
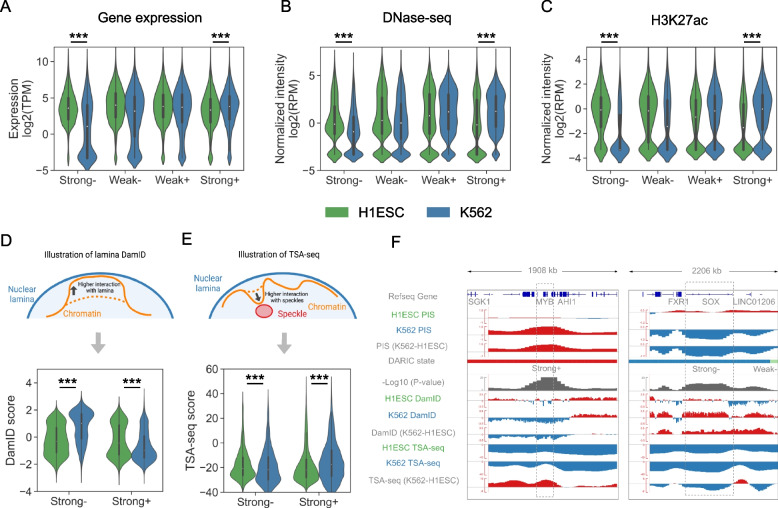
Integration of multi-modal genomics data demonstrates the functional association between gene regulation and differential compartments revealed by DARIC. **A-C** Gene expression (**A**), DNase-seq chromatin accessibility (**B**), and H3K27ac intensity. (**C**) comparison between H1ESC and K562 cells in the four states. ***, *p* < 1e-3, Mann–Whitney U test. TPM, Transcript Per Million. RPM, Reads Per Million mapped reads. **D-E** Illustration and comparison of the lamina B1 DamID signals (**D**) and TSA-seq signals. **E** between H1ESC and K562 cells in the PIS strongly changed regions. **F** Snapshots at the *MYB* and *SOX2* gene loci showing the PIS comparison between H1ESC and K562 cells, as well as the concordant changes in DamID and TSA-seq signals

To test whether the quantitatively differential compartmentalization analysis provided by DARIC increases the power for identifying functional relevant changes, we interrogated the transcriptomic and epigenetic data to see if concordant changes are observed in regions with significantly changed PIS. Using the comparison between H1ESC and K562 cells, we evaluated the changes in gene expression from RNA-seq, chromatin accessibility from DNase-seq, and H3K27ac distribution from ChIP-seq. Indeed, consistent changes were observed in all the three modalities, where genomic regions with strongly decreased PIS in K562 cells (i.e. “Strong-”) are associated with significantly lower gene expression, chromatin accessibility, and H3K27ac signals (Fig. 2A, B and C). Opposite trends were also observed in the “Strong + ” state, suggesting that higher PIS correlates with higher transcription and a more active chromatin state. To test whether PIS changes are associated with functionally important genome regions, we performed enrichment analysis of cell-type-specific genes, as well as super-enhancers, in the four types of domains. The rationale for using super-enhancers is that super-enhancers are hallmarks of cell fate and cell identity [38]. As shown in Figure S2A and B, cell-type-specific genes and super-enhancers are most significantly enriched in the genomic regions with strongly changed PIS and moderately enriched in domains with weakly changed PIS. Taken together, our analyses demonstrated that the quantitative compartmentalization changes are closely related to gene regulation and play a critical role in cell identity.

Many genome-wide techniques have emerged recently to probe the positioning of chromosomal regions with respect to specific subnuclear structures, such as speckles and laminas, and revealed that differential nuclear positioning adds an additional layer of regulation to modulate gene expression [11, 12, 16, 17]. To show if the PIS changes derived from Hi-C data correlate with the differences in nuclear positioning, we integrated the lamina B1 DamID and TSA-seq data in H1ESC and K562 cells from the 4D Nucleosome consortium [13]. Specifically, lamina B1 DamID is a technique to measure the physical interactions between chromosomal regions and the lamina B1 protein in nuclear membrane. Higher DamID signals represent higher interactions between chromatin and lamina [12] (Fig. 2D). TSA-seq estimates the cytological distance between chromosomal regions and nuclear speckles. A higher TSA-seq score means a closer distance to speckles and is usually associated with higher transcriptional activities and more active chromatin states [17] (Fig. 2E). After normalization, our data show that regions with strongly increased PIS (“Strong + ”) in K562 cells have lower interactions with the nuclear lamina and closer distance to nuclear speckles. A consistent trend was also observed for regions with decreased PIS (“Strong-”). Figure 2F summarizes the close relation between PIS changes and the other two techniques by using two cell-type marker genes, *MYB* and *SOX2*. *MYB* is a transcription factor that plays an essential role in hematopoiesis [39] and is highly expressed in K562 cells (Figure S2C), whereas *SOX2* is a critical regulator related to pluripotency [40] and highly expressed in H1ESCs (Figure S2C). At the *MYB* locus, DARIC reveals that there is a significant increase in PIS in K562 cells as compared to H1ESC. In concordance, the interaction between the *MYB* locus and nuclear lamina is significantly reduced and the distance to nuclear speckles is closer. Trends with consistent changes were also seen for the *SOX2* locus. Taken together, our analyses suggest that the quantitative differences in PIS may provide a useful guide for the investigation of nuclear repositioning in development and diseases.

### Comparison between DARIC and existing methods

Unlike the conventional compartment switching analysis which focuses on the qualitative differences, DARIC adopts a quantitative approach. To demonstrate DARIC’s improvements over the conventional method, we compared these two approaches in the differential compartment analysis between H1ESC and K562 cells.

**Fig. 3:**
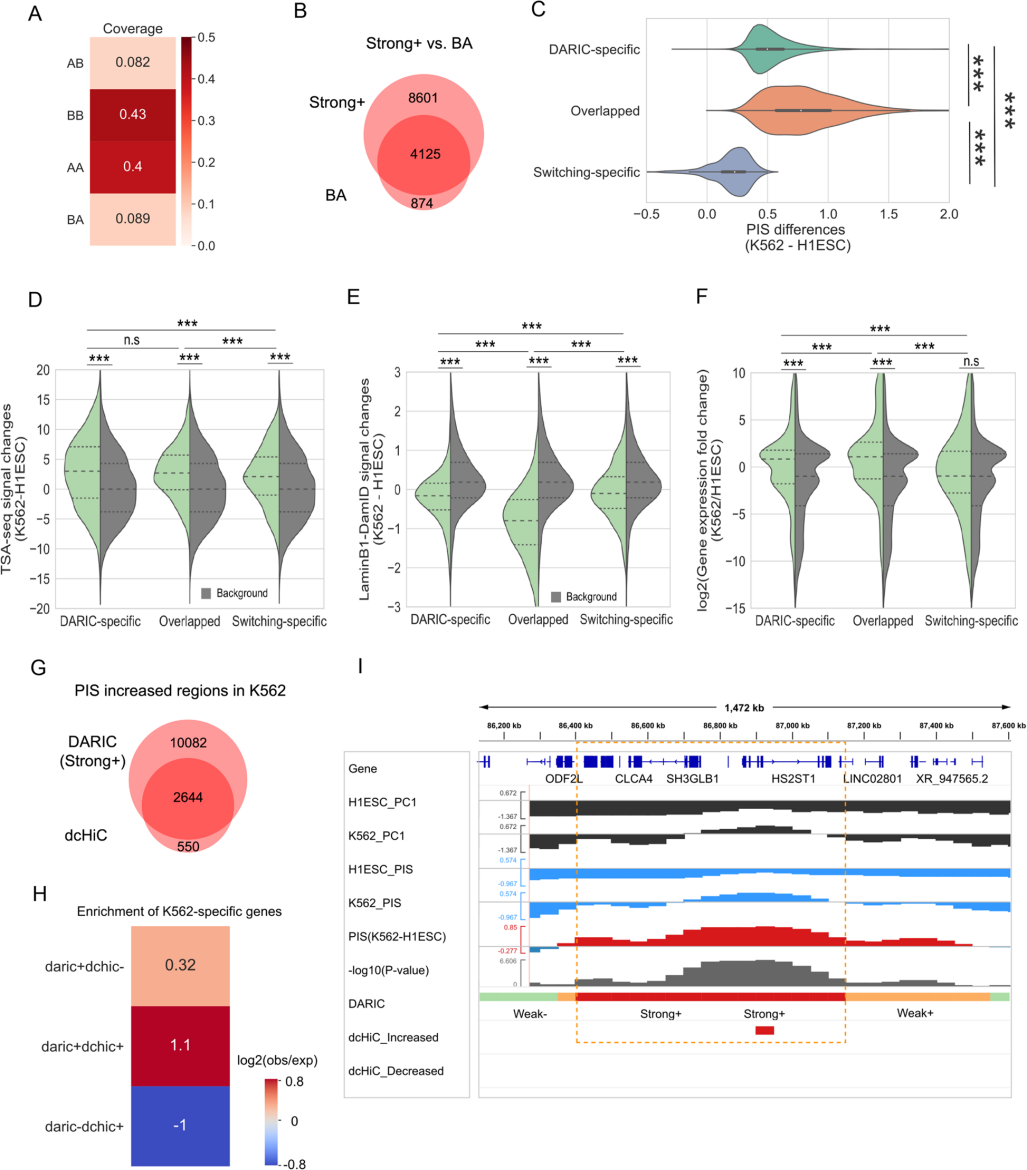
Comparison between DARIC and existing methods. **A** Genomic coverage of the four types of domains in terms of compartment changes. ‘AB’ represents a 50 kb genomic bin in compartment A in H1ESC that switch to compartment B in K562 cells. **B** Venn diagram representing the overlap between the ‘Strong + ’ state revealed by DARIC and the ‘BA’ state in PC1-based switching analysis. The numbers in the plot represent the numbers of 50 kb bins. **C** Violin plot showing the PIS differences for the three types of domains defined in (**B**). **D-F** Violin plots showing the comparisons of TSA-seq signal changes (**D**), lamina B1 DamID signal changes (**E**), and gene expression fold changes (**F**) in the three types of domains defined in (**B**). Gray distributions in each figure represent the signal changes for background regions that are randomly selected from the genome.***, *p* < 1e-3;n.s, not significant (*p* > 0.05), Mann-Whitney U test. **G** Venn diagram showing the overlap of genomic bins identified with increased PIS/PC1 values in K562 by DARIC and dcHiC. Numbers of 50 kb bins were shown in the diagram. **H** Enrichment of K562-specific genes for the three types of genomic regions defined in (**G**). **I** An exemplary region showing DARIC and dcHiC output with increased PIS in K562 cells

To this end, we first performed PCA in H1ESC and K562, each with two replicates. The PC1 values from the two replicates were averaged within each cell type and then compared between the two cell types, which revealed four types of genomic bins: AA and BB (bins without switching compartments), AB, and BA (bins switched compartments). As shown in Fig. 3A, 8.2% of the genome switch from compartment A to B, and 8.9% of the genome is characterized by the opposite switch, much less than the genomic coverage revealed by the quantitative analysis by DARIC (Fig. 1E). Furthermore, we overlapped the PC1-switched genomic bins with the quantitatively differential bins revealed by DARIC. Notably, most of the genomic bins switching from compartment B to A (4125 out of 4999, 83%) are identified by DARIC (Fig. 3B). A comparison of PIS differences reveals that the 874 switching-specific genomic bins (17% of the total “BA” genomic bins) have significantly lower differences than the other types of regions, explaining why they are not identified by DARIC (Fig. 3C). It was also observed that there are more genomic regions that are characterized by significant quantitative PIS increases without switching compartments (hereafter denoted as DARIC-specific regions) (Fig. 3B). To test whether these loci represent functionally relevant changes, we evaluated the TSA-seq, DamID, and gene expression changes among the three types of regions: DARIC-specific, overlapped, and switching-specific, with the random genomic regions used as background. Compared to the random background, DARIC-specific regions have higher TSA-seq signals (Fig. 3D), lower DamID signals (Fig. 3E), and higher gene expressions (Fig. 3F), suggesting that those regions unique to the quantitative analysis are functionally important and thus cannot be overlooked in the differential compartment analysis. Similarly, we performed the analysis between the quantitatively decreased regions (“Strong-”) with genomic bins switched from A to B (i.e. “AB”), and obtained results showing the same trend (Figure S3A-D). Collectively, by comparing DARIC with the prevalent compartment switching analysis, we showed that the conventional switching analysis misses a significant portion of quantitatively differential genomic regions which are closely associated with differential nuclear positioning and gene expression.

Next we further compared DARIC with dcHiC [41], a recent method using the quantitative PC1 values to find the differential compartmentalization domains. We applied dcHiC to H1ESC and K562 cells and identified the significantly differential genomic bins at a bin resolution of 50 kb and a p-value cut-off of 0.01 (same as the cutoff used in DARIC). Subsequently, the genomic bins identified with significantly increased PC1 values in K562 were compared with the PIS increased regions (i.e. “Strong + ”) from DARIC. As shown in Fig. 3G, most of the genomic bins identified by dcHiC (83%, 2644 out of 3194 bins) are also identified by DARIC. To check if the genomic regions specific to each method are biologically relevant, we looked at if K562-specific genes are enriched in those regions. A higher enrichment of K562-specific genes would suggest a tighter functional relevancy for the genomic regions. Figure 3H shows that the regions identified by DARIC are enriched with K562-specific genes, suggesting DARIC-specific genomic regions are biologically meaningful. As expected, the regions identified by both methods exhibit the highest enrichment of K562-specific genes. Nevertheless, dcHiC-specific regions did not demonstrate an enrichment of K562-specific genes. The same analyses were also repeated for PIS-decreased regions and similar conclusions were reached (Figure S3E, F). Notably, compared with dcHiC which tend to output discrete and discontinuous genomic bins as results (Fig. 3I and Figure S3G), DARIC can output the whole differential domain and keep the domain continuity nature of chromatin, largely due to the usage of HMM model which considers effects of neighboring chromatin regions. We also compared DARIC with HOMER, a method to find differential compartment domains based on the correlation between interaction profiles of two Hi-C matrices. Similar results were obtained (Figure S3H-K). Altogether, these results strongly suggest that DARIC achieves superior performance in the comparative analyses of compartmentalization.

### DARIC is robust to protocol and technical variations of Hi-C

A common challenge in Hi-C data comparison relates to the protocol and technical variations involved in data generation, such as the choice of restriction enzymes and sequencing depth. Indeed, a systematic evaluation of chromosome conformation assays by Oksuz et al. [42] reveals that the usage of different restriction enzymes can affect compartment analysis. As such, it is critical to assess and remove these systematic biases before quantitative and statistical analyses.

**Fig. 4:**
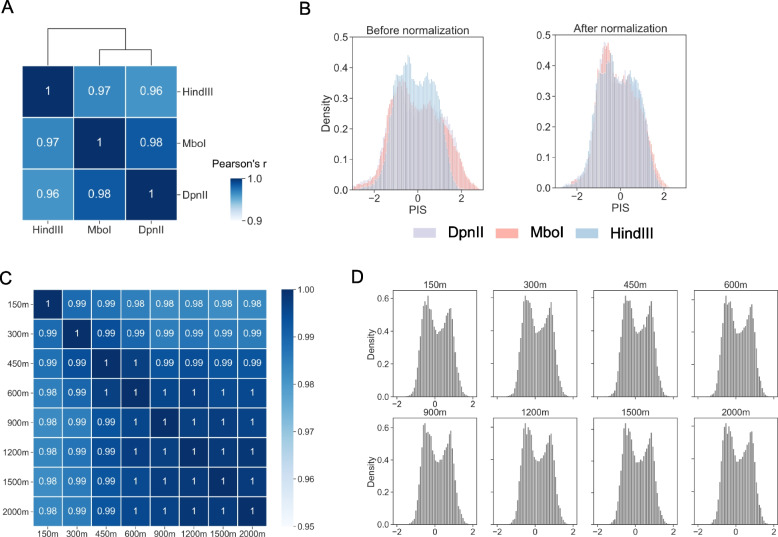
DARIC is robust to technical variations in Hi-C data, such as choices of restriction enzymes and sequencing depth. **A** Heatmap showing the high correlation between PIS tracks generated from Hi-C data of different restriction enzymes, including HindIII, MboI, and DpnII. The three datasets are from GM12878. **B** Histograms showing the distributions of PIS calculated from Hi-C datasets with the three restriction enzymes, before and after normalization by DARIC. **C** Heatmap showing the high correlation of PIS calculated from Hi-C datasets at different sequencing depths. **D** Histograms showing the similar distributions of PIS from Hi-C data at different sequencing depths

To assess the effects of restriction enzymes on differential compartment analysis, we calculated and compared the PIS tracks of Hi-C samples from the same cell type and with three restriction enzymes that are commonly used in Hi-C protocols: HindIII, MboI, and DpnII. While MboI and DpnII produce fragments at kilobase resolution, HindIII cuts the genome at relatively large fragments of several kilobases. Specifically, the three Hi-C datasets generated in GM12878 cells were downloaded from the 4DN consortium. They were down-sampled to the same sequencing depth and PIS tracks were calculated. A comparison of PIS tracks reveals that they are highly correlated, while MboI and DpnII PIS tracks have a slightly higher correlation, likely because the two enzymes recognize and cut the same DNA sequences (Fig. 4A). However, visualization of PIS tracks in the genome browser (Figure S4A, upper panel) or examination of the PIS distribution (left panel of Fig. 4B) reveals that HindIII is distinct from the other two in data scaling, where HindIII dataset displays lower amplitude, suggesting the necessity to remove this systematic difference before further analysis. We used the MA-norm module from DARIC to normalize the MboI and DpnII PIS tracks with respect to HindIII and observed that the distinct differences in data scaling are eliminated, as shown in Fig. 4B (right panel) and Figure S4A (lower panel), suggesting that DARIC can robustly handle Hi-C datasets from different restriction enzymes.

Sequencing depth is another common yet crucial confounding factor in Hi-C data comparison. To assess the impacts of sequencing depth on DARIC, we compared the PIS tracks resulting from Hi-C datasets at various sequencing depths. Specifically, we downloaded a deeply sequenced Hi-C dataset of H1ESC from the 4DN consortium and down-sampled it into eight different depths, ranging from 150 million to 2 billion valid read pairs. PIS tracks were then obtained from DARIC at the resolution of 50 kb. Similarity analysis reveals that all the PIS tracks are highly correlated (Fig. 4C). Furthermore, PIS distributions (Fig. 4D), as well as genome browser snapshots (Figure S4B), show that these Hi-C datasets at different sequencing depths display no obvious differences in data scaling, suggesting that DARIC is not sensitive to the sequencing depth of the Hi-C data. Taken together, our analyses demonstrate that DARIC is robust to protocol and technical variations of the input Hi-C data.

### Applying DARIC to time-course Hi-C data during cardiomyocyte differentiation reveals that activated genes involving compartmentalization changes correspond to more specific cellular functions

**Fig. 5:**
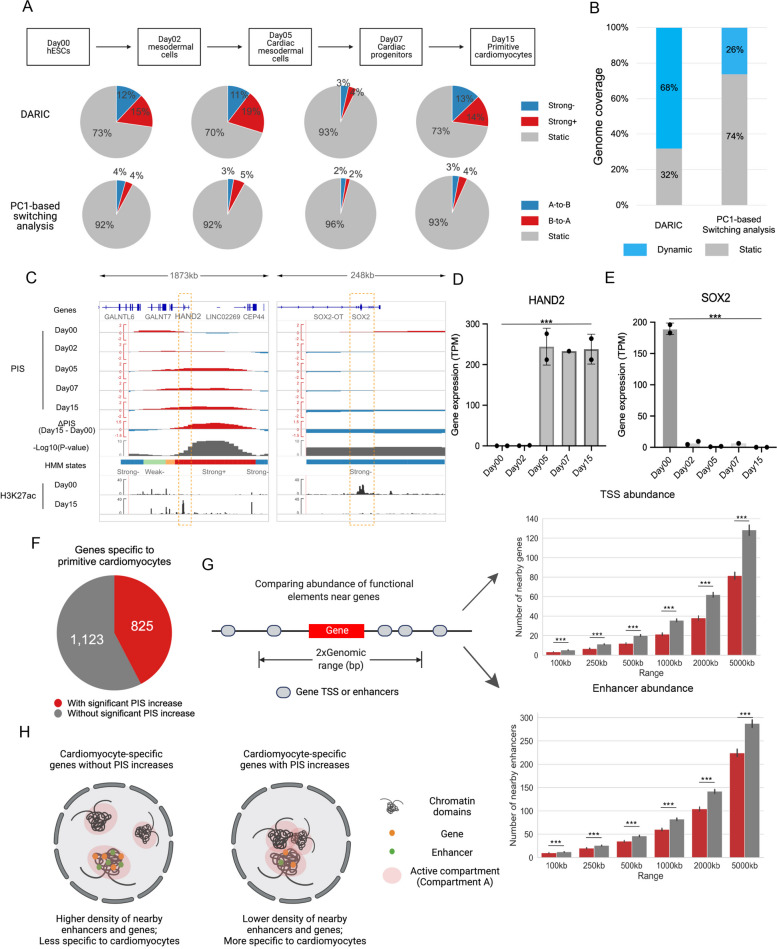
Applying DARIC to delineating compartment changes during cardiomyocyte differentiation. **A** Flow charts and pie charts comparing the genomic coverage of significantly changed regions in compartmentation revealed by DARIC and PC1-based switching analysis. **B** Stacked bar plot showing the percentage of genomic regions undergoing significant compartment changes during the cardiomyocyte differentiation. **C** Genome browser snapshots at the *HAND2* and *SOX2* locus showing compartment changes during differentiation and concordant epigenetic changes. **D**, **E** Bar plots showing the concordant gene expression changes for *HAND2* (**D**) and *SOX2* (**E**) during the differentiation. ***, *p* < 0.001, Wald test from DESeq2. **F** Pie chart representing the numbers of cardiomyocyte-specific genes which are associated or not associated with significant PIS increases. Cardiomyocyte-specific genes are defined as those significantly up-regulated in Day15 compared to Day00. **G** Gene and enhancer density comparison for the two gene sets as defined in (**F**). **H** An illustration showing the two mechanisms involved in the activation of cardiomyocytespecific genes during differentiation

In order to systematically characterize dynamic compartmentalization landscapes during cell differentiation, we applied DARIC to a time-course Hi-C dataset [33] delineating the human cardiomyocyte differentiation from ESCs at day 0 to primitive cardiomyocytes at day 15, with three intermediate stages (top panel of Fig. 5A). Specifically, PIS tracks were calculated from the Hi-C data at each time point and residual PIS tracks were obtained for neighboring time points. DARIC was then applied to the four residual PIS tracks simultaneously to identify the genomic regions with significant compartmentalization changes. A single HMM model was trained for all the samples to facilitate cross-sample comparison (Figure S5A). Comparing DARIC’s results with PC1-based switching analysis, as expected, we found a much higher percentage of the genome associated with significant changes in compartmentalization (Fig. 5A). For example, during the transition from Day00 to Day02, DARIC identifies ~ 4 times more genomic regions with strongly increased compartmentalization with active compartment A than the conventional PC1-based switching analysis (15% vs. 4%). Furthermore, in the whole differentiation course, 68% of the genome is associated with compartmentalization changes, yet only 26% of the genome has switched compartments (Fig. 5B), suggesting that the plasticity of the compartmentalization landscape was greatly underestimated by the PC1-based switching analysis. Among the genomic regions with significant quantitative changes in compartmentalization, there are genes with critical roles in regulating cell identity, such as *HAND2* and *SOX2* (Fig. 5C). *HAND2* is an essential transcription factor for cardiac morphogenesis [43] and is up-regulated during cardiomyocyte differentiation (Fig. 5D). Temporal PISs show that the *HAND2* locus has increasingly higher interactions with the active compartment A and DARIC correctly annotates the locus with “Strong + ” when comparing Day15 with Day00. A similar example was for *SOX2*, which is a critical factor in pluripotency [44] and is associated with gradually decreasing interactions with the active compartment during cardiomyocyte differentiation (Fig. 5C and E). Collectively, these analyses indicate that the quantitative compartmentalization changes are important for cell identity and DARIC can accurately capture these changes.

Out of the 1948 genes that are activated in Day15 comparing to Day00, 825 genes (42%) are associated with significant PIS increases (Fig. 5F). We speculated that there might be differences in the activation mechanisms of these two sets of genes. Indeed, we found that the genes involved in significant PIS increases tend to be less accessible to local functional elements like genes or enhancers because they tend to reside in less gene-dense or enhancer-dense regions (Fig. 5G). We hypothesize that the activity of these genes are more preferentially regulated via large-scale 3D genome reorganization (Fig. 5H). As additional support, we analyzed the corresponding Hi-C data and observed that the chromatin loops associated with the genes involved in PIS increases are significantly longer (Figure S5B). Gene Ontology enrichment analysis suggests that the genes associated with PIS increases are more enriched in cardiomyocyte-specific functions (Figure S5C), consistent with previous studies showing that more specific genes tend to use distant regulatory elements [45, 46]. Taken together, our analyses using DARIC reveal more biological insights about the dynamic compartmentalization during cell differentiation.

### Evaluating compartmentalization variability landscape of human genome 

**Fig. 6:**
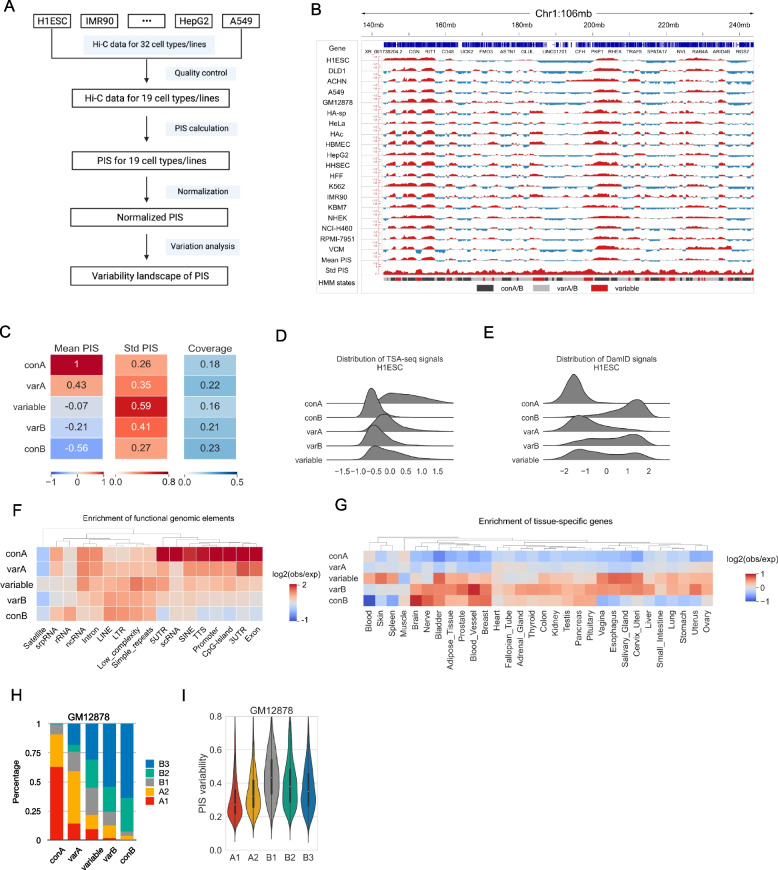
Applying DARIC to a compendium of Hi-C datasets across many cell types. **A** Flowchart showing the pipeline of the analysis. **B** A snapshot of chromosome 1 showing the PIS tracks across 19 cell lines, the mean and standard deviation of PIS, and segmentation of the genome into five states according to the mean and variability of PIS. **C** Mean and standard deviation of PIS, as well as the genomic coverage, for the five HMM states. **D**, **E** Distributions of TSA-seq (**D**) and DamID signals (**E**) for the five types of states. **F** Heatmap depicting the enrichment of functional genomic elements in the five types of domains. **G** Heatmap showing the enrichment of tissue-specific genes in the five types of domains. **H** Stacked bar plot showing the sub-compartment composition for the five types of domains. **I** PIS variability, defined as the standard deviation of PIS across the 19 cell lines, of the five types of sub-compartments

A thorough understanding of the compartmentalization variability landscape of the human genome is still lacking, largely due to the limitations in the qualitative switching-based analysis. The compartmentalization quantification and normalization modules in DARIC provide an unbiased approach for this task. To this end, we leveraged the rich Hi-C resources housed in the 4DN consortium and evaluated the variability landscape of compartmentalization of the human genome (Fig. 6A). We first performed a comprehensive query and interrogation of available Hi-C datasets in 4DN, resulting in Hi-C data of 32 cell lines or primary cells without additional treatments. We analyzed the compartmentalization landscape at the resolution of 50 kb. Quality control filtered out 13 Hi-C samples likely due to limited sequencing depth, resulting in Hi-C data for 19 cell types, covering blood cells (B lymphocytes, lymphoblasts), various brain cells (astrocytes of the cerebellum/spinal cord, and microvascular endothelial cells), lung epithelial cells, skin malignant melanoma and others (see detailed information of all the 19 Hi-C datasets in Table S2). We then calculated PIS for each cell type and normalized it with respect to the PIS in H1ESC. The normalized PIS tracks were then used for the variability analysis (Fig. 6B).

We used the standard deviation of PIS as the metric to assess the compartmentalization variability. To identify the genomic domains at different variability levels, we trained an HMM model based on the mean and standard deviation of PIS to segment the genome into five types of domains: “conserved A” (con A), “conserved B” (con B), “variable A” (var A), “variable B” (var B), and “variable” (Fig. 6B and C), where “con A” and”con B” represent the domains in constitutive compartment A or B and show low variability, “variable A” and “variable B” represent domains with slightly higher variability but a clear compartmental type preference, and “variable” domains display the highest variability level and show frequent switching between the two compartment types (Fig. 6C). Nuclear positioning data in H1ESCs, such as TSA-seq and lamina B1 DamID data, reveal that these five states occupy distinct spaces in the lamina-to-speckle axis, where “conA” and “con B” regions are the most distant or closest to nuclear lamina respectively, and the other three states exhibit a gradual trend in the intermediate space (Fig. 6D and E). Same trends were observed in other cell types (Figure S6A and B). Furthermore, the five states display gradually differential enrichments in genomic elements (Fig. 6F), where the conA state is the most enriched in CpG-islands, promoters, and UTRs, and the conB state shows the strongest depletion in these states. Elements with a high level of sequence repeating, such as LINEs (Long Interspersed Nuclear Elements), LTRs (Long Terminal Repeats), and simple repeats, tend to show higher enrichment in non-A-type compartments (i.e. variable, varB and conB). To understand if there is a link between compartmentalization variability and gene expression variability, we looked at how cell-type-specific genes distribute in the five states with distinct compartment variability (Fig. 6G). To this end, we accessed the gene expression matrix spanning 30 tissues from GTEx [47] and examined the enrichment of tissue-specific genes in the five states. Specifically, we used Tau index [48], which ranges from 0 (uniformly expressed in all tissues) to 1 (uniquely expressed in one tissue, see methods for details), to measure each gene’s expression specificity. Genes with a Tau index higher than 0.3 were deemed as specific genes in one tissue. Interestingly, we found that tissue-specific genes are highly enriched in the non-A-type state (Fig. 6G), and the variable state exhibits an overall strong enrichment across the panel of tissues, highlighting a strong correlation between compartment variability and expression specificity.

Sub-compartments are finer chromatin structures that can be obtained from Hi-C data with high coverage. Analysis in GM12878 cells [6] revealed that compartment A can be divided into two sub-compartments, A1 and A2, and compartment B into three sub-compartments, B1-3. Sub-compartments exhibit refined associations with many functional features, such as histone modification marks, replication timing, and gene expression [6]. However, a thorough understanding of sub-compartments is still lacking. Our analysis in the compendium of Hi-C datasets enables a better understanding of the compartmentalization variability at the sub-compartment level. Notably, we found that the five states based on compartment variability show a remarkable correspondence to sub-compartments in GM12878 data, where the conA state is mostly composed of the active A1 state and the conB state is mostly made up of inactive B3 states (Fig. 6H). A direct comparison of the compartment variability of the five types of sub-compartments further supports that there is a differential pattern in the variability of the five sub-compartments (Fig. 6I). Similarly, results in other cell types reveal the same observation (Figure S6C and D). Taken together, our analyses strongly suggest that compartment variability corresponds well with the sub-compartments.

## Discussion

While PCA is a powerful approach for binary classification of the genome into the active and inactive compartments, PC1-based switching analysis is insufficient to reflect the true changes in compartmentalization due to its inherent qualitative nature. To bridge this gap, we developed DARIC, a computational framework to find the quantitatively differential compartment domains. We developed a metric named PIS to represent the preference of compartment type and quantify the interaction strength with the active A compartment relative to the B compartment. Remarkably, although PIS shows a high correlation with the PC1 values from the PCA analysis, it has several advantages. For example, PIS has a clear biological meaning, thus enabling further normalization and statistical comparison analysis. Comparison of Hi-C datasets is usually complicated by the technical and protocol variations. To overcome this challenge, we borrowed a concept from MA-norm, a powerful method originally developed for the normalization of ChIP-seq data, for normalizing the PIS of two Hi-C samples. We demonstrated that this normalization module can eliminate the systematic bias between two Hi-C datasets, thus enabling a fair and robust differential analysis. To find the differential compartment domains, we used HMM to segment and annotate the genome into four states, where the domains with strongly increased or decreased PIS are identified as the differential ones. If replicate data are available, DARIC can perform further statistical analyses to evaluate the significance of the differential domains, to filter out noises caused by technical variations. In summary, DARIC provides a complete framework and pipeline for quantitatively differential compartment analyses.

We integrated transcriptomics, chromatin accessibility, and epigenetic profiling data to validate the functional relevance of the quantitatively differential compartmental domains identified by DARIC. Our analyses demonstrate that quantitative PIS changes correspond to concordant changes in these modalities. Furthermore, integrative analysis with nuclear positioning data shows that PIS changes correlate with the differential nuclear positioning in the lamina-to-speckle axis, suggesting that PIS changes can be used to estimate nuclear positioning differences with chromatin conformation data as the input. Altogether, our analyses demonstrate that DARIC effectively identifies quantitative and biologically meaningful changes in chromatin states and nuclear positioning, which could not be captured by the previous method [4].

We illustrated the utility of DARIC by applying it to delineate the compartmentalization dynamics in cardiomyocyte differentiation and analyzing the compartmentalization variability landscape using a compendium of Hi-C data. Compared to the conventional PC1-based switching analyses, DARIC identified significantly more genomic regions undergoing compartmental changes during differentiation, which include genes that are critical for cell identities, such as *HAND2* and *SOX2* genes during cardiomyocyte differentiation from ESCs. These findings further highlight the importance of the quantitative compartmentalization changes and suggest that the degree of compartmentalization plasticity is underestimated by previous analyses. We further found that the activated genes with significant PIS increases are more specific and less abundant in local functional elements like genes and enhancers, in line with the previous findings that more specific genes are more involved in interactions with more distal elements. Those applications demonstrate that DARIC is useful in identifying the differential compartmental regions and revealing new insights into how 3D genome organization adds an additional layer to gene regulation.

### Supplementary Information

**Additional file 1: Figure 1. **Introduction of the DARIC framework. A Scatter plot showing the high correlation between PIS and PC1 values from the H1ESC Hi-C data. B MA plot showing the systematic differences between H1ESC and K562 cells. Each dot represents a 50kb bin. The Red dashed line represents the fitted line from the M and A values. C MA plot after normalization showing the elimination of the systematic differences between the two cell types. D-E The emission matrix (D) and state coverage matrix (E) for the 5-state HMM model. F Confusion matrix showing the overlap between the states of 5-state model and those of the 4-state model. Numbers represent 50kb bins. **Figure 2.** Functional association between gene regulation and differential compartments revealed by DARIC. A-B Heatmap showing the enrichment of cell type-specific genes (A) and superenhancers. (B) in the four states identified by DARIC. Values show the log2(observed/expected) enrichment. C Bar plots showing the expression of SOX2 and MYB genes in H1ESC and K562 cells. **Figure 3.** Comparison between DARIC and existing methods. A Venn diagram presenting the overlap between the ‘Strong-’ state revealed by DARIC and the ‘AB’ state in conventional analyses. The numbers in the plot represent the numbers of 50kb bins. B Violin plot showing the PIS differences for the three types of domains defined in (A). C-D Violin plots showing the comparisons of Lamina1-DamID signal changes (C), and gene expression fold changes (D) in the three types of domains defined in (A). E Venn diagram showing the overlap of genomic bins identified with decreased PIS/PC1 values in K562 by DARIC and dcHiC. Numbers of 50kb bins were shown in the diagram. F Enrichment of H1ESC-specific genes for the three types of genomic regions defined in (E). G An exemplary region showing DARIC and dcHiC output with decreased PIS in K562 cells. H-K Performance comparison between DARIC and HOMER using H1ESC versus K562 as an example. (H) Venn diagram showing the overlap of genomic bins identified with increased PIS values in K562 by DARIC and HOMER. Numbers of 50kb bins were shown in the diagram. (I) Enrichment of K562-specific genes for the three types of genomic regions defined in (H). (J) Venn diagram showing the overlap of genomic bins identified with decreased PIS values in K562 by DARIC and HOMER. Numbers of 50kb bins were shown in the diagram. (K) Enrichment of H1ESC-specific genes for the three types of genomic regions defined in (J). **Figure 4.** DARIC is robust to technical variations in Hi-C data, such as choices of restriction enzymes and sequencing depth. A Snapshot of chromosome 6 showing the comparison in scaling differences in PIS from three different restriction enzymes before and after the normalization step performed by DARIC. B Snapshot of chromosome 6 showing the high similarity of PIS from Hi-C data at different sequencing depths. **Figure 5.** Applying DARIC to delineating compartment changes during cardiomyocyte differentiation. A Emission matrix resulting from the HMM model trained in the cardiomyocyte system. B Cardiomyocyte-specific genes associated with significant PIS increases during the differentiation tend to be involved in longer loops than those without PIS increases. C GO enrichment analysis for two sets of cardiomyocyte-specific genes classified by whether associated with significant PIS changes. **Figure 6.** Applying DARIC to a compendium of Hi-C datasets across many cell types. A Distribution of TSA-seq signals in the five variability states in the three cell lines. B Distribution of DamID signals in K562 cells. C Stacked bar plots showing the composition percentages of the five sub-compartments in the five variability states. D PIS variability comparison for the five sub-compartments.

**Additional file 2: Table S1.** Data used for the H1ESC-K562 comparison.

**Additional file 3: Table 2.** Analyzed Hi-C datasets from the 4DN consortium.

## Data Availability

All datasets, including Hi-C data and functional genomics data, used in H1ESC vs. K562 comparison are summarized in Table S1. Hi-C datasets used for the compartmentalization variability analysis were summarized in Table S2. PIS tracks for a compendium of cell types in 4DN, as well as the PIS variability track and the domains at differential variability levels identified in this study, are publicly available in https://github.com/ykai16/DARIC/tree/main/data. DARIC is freely available at Github (https://github.com/ykai16/DARIC) and Pypi (https://pypi.org/project/daric/).

## References

[1] Szabo Q, Bantignies F, Cavalli G: Principles of genome folding into topologically associating domains. Sci Adv 2019, 5(4):eaaw1668. 10.1126/sciadv.aaw1668 PMC6457944 30989119

[2] Gibcus JH, Dekker J (2013). The hierarchy of the 3D genome. Mol Cell.

[3] Bonev B, Cavalli G (2016). Organization and function of the 3D genome. Nat Rev Genet.

[4] Bhat P, Honson D, Guttman M (2021). Nuclear compartmentalization as a mechanism of quantitative control of gene expression. Nat Rev Mol Cell Biol.

[5] Lieberman-Aiden E, van Berkum NL, Williams L, Imakaev M, Ragoczy T, Telling A, Amit I, Lajoie BR, Sabo PJ, Dorschner MO (2009). Comprehensive mapping of long-range interactions reveals folding principles of the human genome. Science.

[6] Rao SS, Huntley MH, Durand NC, Stamenova EK, Bochkov ID, Robinson JT, Sanborn AL, Machol I, Omer AD, Lander ES (2014). A 3D map of the human genome at kilobase resolution reveals principles of chromatin looping. Cell.

[7] Hsieh TS, Cattoglio C, Slobodyanyuk E, Hansen AS, Rando OJ, Tjian R, Darzacq X. Resolving the 3D Landscape of transcription-linked mammalian chromatin folding. Mol Cell 2020, 78(3):539–553 e538. 10.1016/j.molcel.2020.03.002 PMC7703524 32213323

[8] Krietenstein N, Abraham S, Venev SV, Abdennur N, Gibcus J, Hsieh TS, Parsi KM, Yang L, Maehr R, Mirny LA et al: Ultrastructural Details of Mammalian Chromosome Architecture. Mol Cell 2020, 78(3):554–565 e557. 10.1016/j.molcel.2020.03.003 PMC7222625 32213324

[9] Marchal C, Sima J, Gilbert DM (2019). Control of DNA replication timing in the 3D genome. Nat Rev Mol Cell Biol.

[10] Kumaran RI, Thakar R, Spector DL (2008). Chromatin dynamics and gene positioning. Cell.

[11] Almonacid M, Terret ME, Verlhac MH (2019). Nuclear positioning as an integrator of cell fate. Curr Opin Cell Biol.

[12] Pindyurin AV, Pagie L, Kozhevnikova EN, van Arensbergen J, van Steensel B (2016). Inducible DamID systems for genomic mapping of chromatin proteins in Drosophila. Nucleic Acids Res.

[13] Dekker J, Belmont AS, Guttman M, Leshyk VO, Lis JT, Lomvardas S, Mirny LA, O'Shea CC, Park PJ, Ren B (2017). The 4D nucleome project. Nature.

[14] Briand N, Collas P (2020). Lamina-associated domains: peripheral matters and internal affairs. Genome Biol.

[15] Chen Y, Zhang Y, Wang Y, Zhang L, Brinkman EK, Adam SA, Goldman R, van Steensel B, Ma J, Belmont AS (2018). Mapping 3D genome organization relative to nuclear compartments using TSA-Seq as a cytological ruler. J Cell Biol.

[16] Girelli G, Custodio J, Kallas T, Agostini F, Wernersson E, Spanjaard B, Mota A, Kolbeinsdottir S, Gelali E, Crosetto N, Bienko M. GPSeq reveals the radial organization of chromatin in the cell nucleus. Nat Biotechnol. 2020;38(10):1184–93. 10.1038/s41587-020-0519-y. 10.1038/s41587-020-0519-y PMC7610410 32451505

[17] Zhang L, Zhang Y, Chen Y, Gholamalamdari O, Wang Y, Ma J, Belmont AS. TSA-seq reveals a largely conserved genome organization relative to nuclear speckles with small position changes tightly correlated with gene expression changes. Genome Res. 2020;31:251–64. 10.1101/gr.266239.120 PMC7849416 33355299

[18] Banani SF, Lee HO, Hyman AA, Rosen MK (2017). Biomolecular condensates: organizers of cellular biochemistry. Nat Rev Mol Cell Biol.

[19] Nichols MH, Corces VG (2021). Principles of 3D compartmentalization of the human genome. Cell Rep.

[20] Dixon JR, Jung I, Selvaraj S, Shen Y, Antosiewicz-Bourget JE, Lee AY, Ye Z, Kim A, Rajagopal N, Xie W (2015). Chromatin architecture reorganization during stem cell differentiation. Nature.

[21] Zhou Y, Petrovic J, Zhao J, Zhang W, Bigdeli A, Zhang Z, Berger SL, Pear WS, Faryabi RB. EBF1 nuclear repositioning instructs chromatin refolding to promote therapy resistance in T leukemic cells. Mol Cell 2022, 82(5):1003–1020 e1015. 10.1016/j.molcel.2022.01.015 PMC8897266 35182476

[22] Ahanger SH, Delgado RN, Gil E, Cole MA, Zhao J, Hong SJ, Kriegstein AR, Nowakowski TJ, Pollen AA, Lim DA (2021). Distinct nuclear compartment-associated genome architecture in the developing mammalian brain. Nat Neurosci.

[23] Vilarrasa-Blasi R, Soler-Vila P, Verdaguer-Dot N, Russinol N, Di Stefano M, Chapaprieta V, Clot G, Farabella I, Cusco P, Kulis M (2021). Dynamics of genome architecture and chromatin function during human B cell differentiation and neoplastic transformation. Nat Commun.

[24] Johnstone SE, Reyes A, Qi Y, Adriaens C, Hegazi E, Pelka K, Chen JH, Zou LS, Drier Y, Hecht V, et al. Large-scale topological changes restrain malignant progression in colorectal cancer. Cell. 2020; 182(6):1474–1489 e1423. 10.1016/j.cell.2020.07.030 PMC7575124 32841603

[25] Heinz S, Benner C, Spann N, Bertolino E, Lin YC, Laslo P, Cheng JX, Murre C, Singh H, Glass CK (2010). Simple combinations of lineage-determining transcription factors prime cis-regulatory elements required for macrophage and B cell identities. Mol Cell.

[26] Durand NC, Shamim MS, Machol I, Rao SS, Huntley MH, Lander ES, Aiden EL (2016). Juicer provides a one-click system for analyzing loop-resolution Hi-C experiments. Cell Syst.

[27] Virtanen P, Gommers R, Oliphant TE, Haberland M, Reddy T, Cournapeau D, Burovski E, Peterson P, Weckesser W, Bright J, et al. SciPy 1.0: fundamental algorithms for scientific computing in Python. Nat Methods. 2020; 17(3):261–272. 10.1038/s41592-019-0686-2 PMC7056644 32015543

[28] Shao Z, Zhang Y, Yuan GC, Orkin SH, Waxman DJ (2012). MAnorm: a robust model for quantitative comparison of ChIP-Seq data sets. Genome Biol.

[29] Moore JE, Purcaro MJ, Pratt HE, Epstein CB, Shoresh N, Adrian J, Kawli T, Davis CA, Dobin A, Consortium EP (2020). Expanded encyclopaedias of DNA elements in the human and mouse genomes. Nature.

[30] Liao Y, Smyth GK, Shi W (2014). featureCounts: an efficient general purpose program for assigning sequence reads to genomic features. Bioinformatics.

[31] Love MI, Huber W, Anders S (2014). Moderated estimation of fold change and dispersion for RNA-seq data with DESeq2. Genome Biol.

[32] Khan A, Zhang X (2016). dbSUPER: a database of super-enhancers in mouse and human genome. Nucleic Acids Res.

[33] Zhang Y, Li T, Preissl S, Amaral ML, Grinstein JD, Farah EN, Destici E, Qiu Y, Hu R, Lee AY (2019). Transcriptionally active HERV-H retrotransposons demarcate topologically associating domains in human pluripotent stem cells. Nat Genet.

[34] Ge SX, Jung D, Yao R (2020). ShinyGO: a graphical gene-set enrichment tool for animals and plants. Bioinformatics.

[35] Kaul A, Bhattacharyya S, Ay F (2020). Identifying statistically significant chromatin contacts from Hi-C data with FitHiC2. Nat Protoc.

[36] Palmer D, Fabris F, Doherty A, Freitas AA, de Magalhaes JP (2021). Ageing transcriptome meta-analysis reveals similarities and differences between key mammalian tissues. Aging (Albany NY).

[37] Xiong K, Ma J (2019). Revealing Hi-C subcompartments by imputing inter-chromosomal chromatin interactions. Nat Commun.

[38] Hnisz D, Abraham BJ, Lee TI, Lau A, Saint-Andre V, Sigova AA, Hoke HA, Young RA (2013). Super-enhancers in the control of cell identity and disease. Cell.

[39] Soza-Ried C, Hess I, Netuschil N, Schorpp M, Boehm T (2010). Essential role of c-myb in definitive hematopoiesis is evolutionarily conserved. Proc Natl Acad Sci U S A.

[40] Niwa H, Ogawa K, Shimosato D, Adachi K (2009). A parallel circuit of LIF signalling pathways maintains pluripotency of mouse ES cells. Nature.

[41] Chakraborty A, Wang JG, Ay F (2022). dcHiC detects differential compartments across multiple Hi-C datasets. Nat Commun.

[42] Akgol Oksuz B, Yang L, Abraham S, Venev SV, Krietenstein N, Parsi KM, Ozadam H, Oomen ME, Nand A, Mao H (2021). Systematic evaluation of chromosome conformation capture assays. Nat Methods.

[43] Russell MW, Kemp P, Wang L, Brody LC, Izumo S (1998). Molecular cloning of the human HAND2 gene. Biochim Biophys Acta.

[44] Takahashi K, Yamanaka S (2006). Induction of pluripotent stem cells from mouse embryonic and adult fibroblast cultures by defined factors. Cell.

[45] Cai W, Huang J, Zhu Q, Li BE, Seruggia D, Zhou P, Nguyen M, Fujiwara Y, Xie H, Yang Z (2020). Enhancer dependence of cell-type-specific gene expression increases with developmental age. Proc Natl Acad Sci U S A.

[46] Kai Y, Li BE, Zhu M, Li GY, Chen F, Han Y, Cha HJ, Orkin SH, Cai W, Huang J (2021). Mapping the evolving landscape of super-enhancers during cell differentiation. Genome Biol.

[47] Consortium GT (2013). The Genotype-Tissue Expression (GTEx) project. Nat Genet.

[48] Yanai I, Benjamin H, Shmoish M, Chalifa-Caspi V, Shklar M, Ophir R, Bar-Even A, Horn-Saban S, Safran M, Domany E (2005). Genome-wide midrange transcription profiles reveal expression level relationships in human tissue specification. Bioinformatics.

