# Supplementary material for: Identifying quantitatively differential chromosomal compartmentalization changes and their biological significance from Hi-C data using DARIC
Source: BMC Genomics. 2023 Oct 13;24:614. doi: 10.1186/s12864-023-09675-w (PMC10571287; doi:10.1186/s12864-023-09675-w)

Supplementary Figure 1. Introduction of the DARIC framework.
A Scatter plot showing the high correlation between PIS and PC1 values from the H1ESC Hi-C data.
B MA plot showing the systematic differences between H1ESC and K562 cells. Each dot represents a 50kb bin. The Red dashed line represents the fitted line from the M and A values.
C MA plot after normalization showing the elimination of the systematic differences between the two cell types.
D-E The emission matrix (**D**) and state coverage matrix (**E**) for the 5-state HMM model.
F Confusion matrix showing the overlap between the states of 5-state model and those of the 4-state model. Numbers represent 50kb bins.

Supplementary Figure 2 Functional association between gene regulation and differential compartments revealed by DARIC.
A-B Heatmap showing the enrichment of cell type-specific genes (**A**) and super-enhancers (**B**) in the four states identified by DARIC. Values show the log2(observed/expected) enrichment.
C Bar plots showing the expression of SOX2 and MYB genes in H1ESC and K562 cells.

Supplementary Figure 3 Comparison between DARIC and existing methods.
A Venn diagram presenting the overlap between the 'Strong-' state revealed by DARIC and the 'AB' state in conventional analyses. The numbers in the plot represent the numbers of 50kb bins.
B Violin plot showing the PIS differences for the three types of domains defined in (**A**).
C-D Violin plots showing the comparisons of Lamina1-DamID signal changes (**C**), and gene expression fold changes (**D**) in the three types of domains defined in (**A**).
E Venn diagram showing the overlap of genomic bins identified with decreased PIS/PC1 values in K562 by DARIC and dcHiC. Numbers of 50kb bins were shown in the diagram.
F Enrichment of H1ESC-specific genes for the three types of genomic regions defined in (**E**).
G An exemplary region showing DARIC and dcHiC output with decreased PIS in K562 cells.
H-K Performance comparison between DARIC and HOMER using H1ESC versus K562 as an example. (**H**) Venn diagram showing the overlap of genomic bins identified with increased PIS values in K562 by DARIC and HOMER. Numbers of 50kb bins were shown in the diagram. (**I**) Enrichment of K562-specific genes for the three types of genomic regions defined in (**H**). (**J**) Venn diagram showing the overlap of genomic bins identified with decreased PIS values in K562 by DARIC and HOMER. Numbers of 50kb bins were shown in the diagram. (**K**) Enrichment of H1ESC-specific genes for the three types of genomic regions defined in (**J**).

Supplementary Figure 4 DARIC is robust to technical variations in Hi-C data, such as choices of restriction enzymes and sequencing depth.
A Snapshot of chromosome 6 showing the comparison in scaling differences in PIS from three different restriction enzymes before and after the normalization step performed by DARIC.

B Snapshot of chromosome 6 showing the high similarity of PIS from Hi-C data at different sequencing depths.

Supplementary Figure 5 Applying DARIC to delineating compartment changes during cardiomyocyte differentiation.
A Emission matrix resulting from the HMM model trained in the cardiomyocyte system.
B Cardiomyocyte-specific genes associated with significant PIS increases during the differentiation tend to be involved in longer loops than those without PIS increases.
C GO enrichment analysis for two sets of cardiomyocyte-specific genes classified by whether associated with significant PIS changes.

Supplementary Figure 6 Applying DARIC to a compendium of Hi-C datasets across many cell types.
A Distribution of TSA-seq signals in the five variability states in the three cell lines.
B Distribution of DamID signals in K562 cells.
C Stacked bar plots showing the composition percentages of the five sub-compartments in the five variability states.
D PIS variability comparison for the five sub-compartments.

Supplementary Figure 1

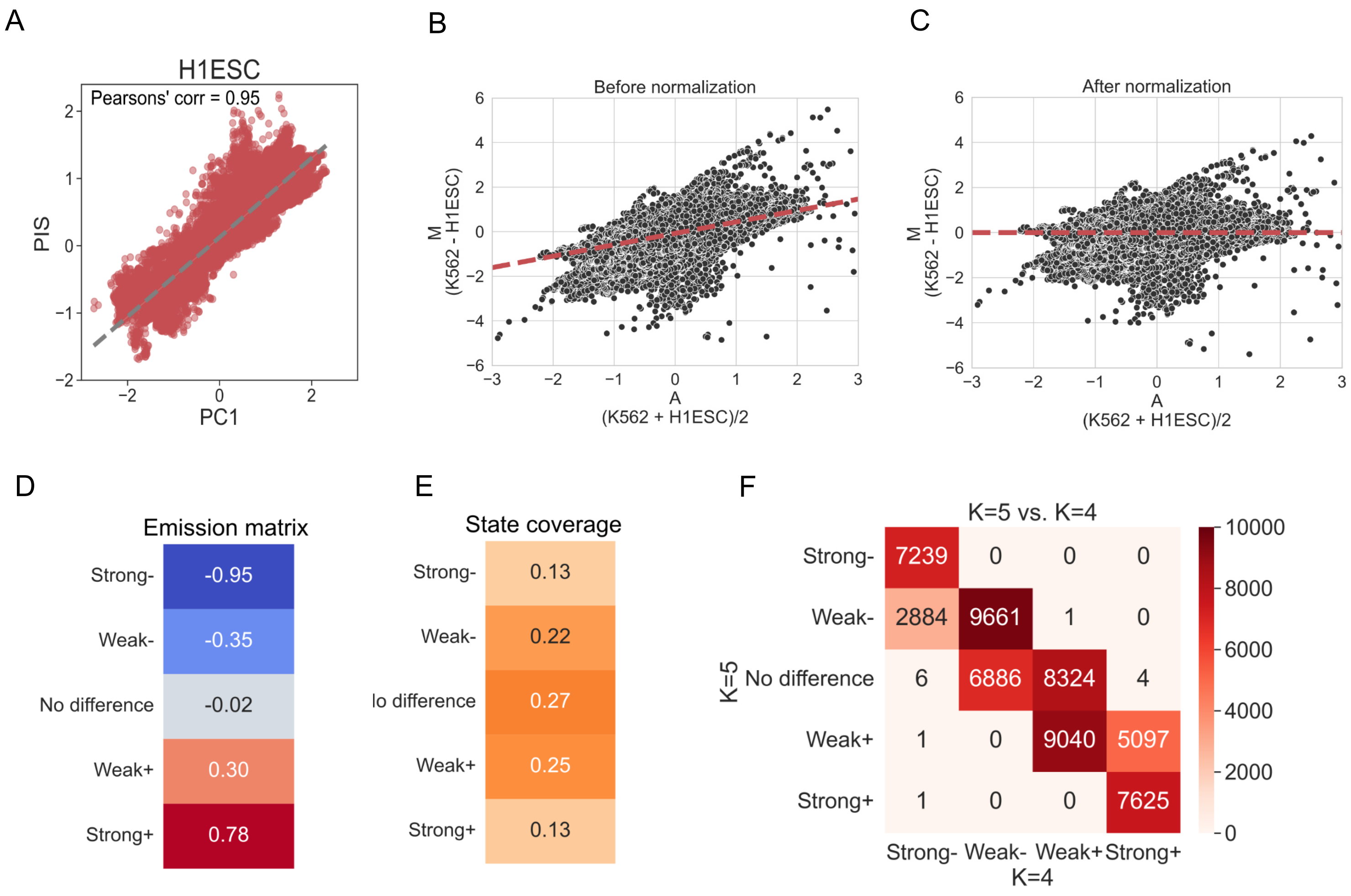

Supplementary Figure 2

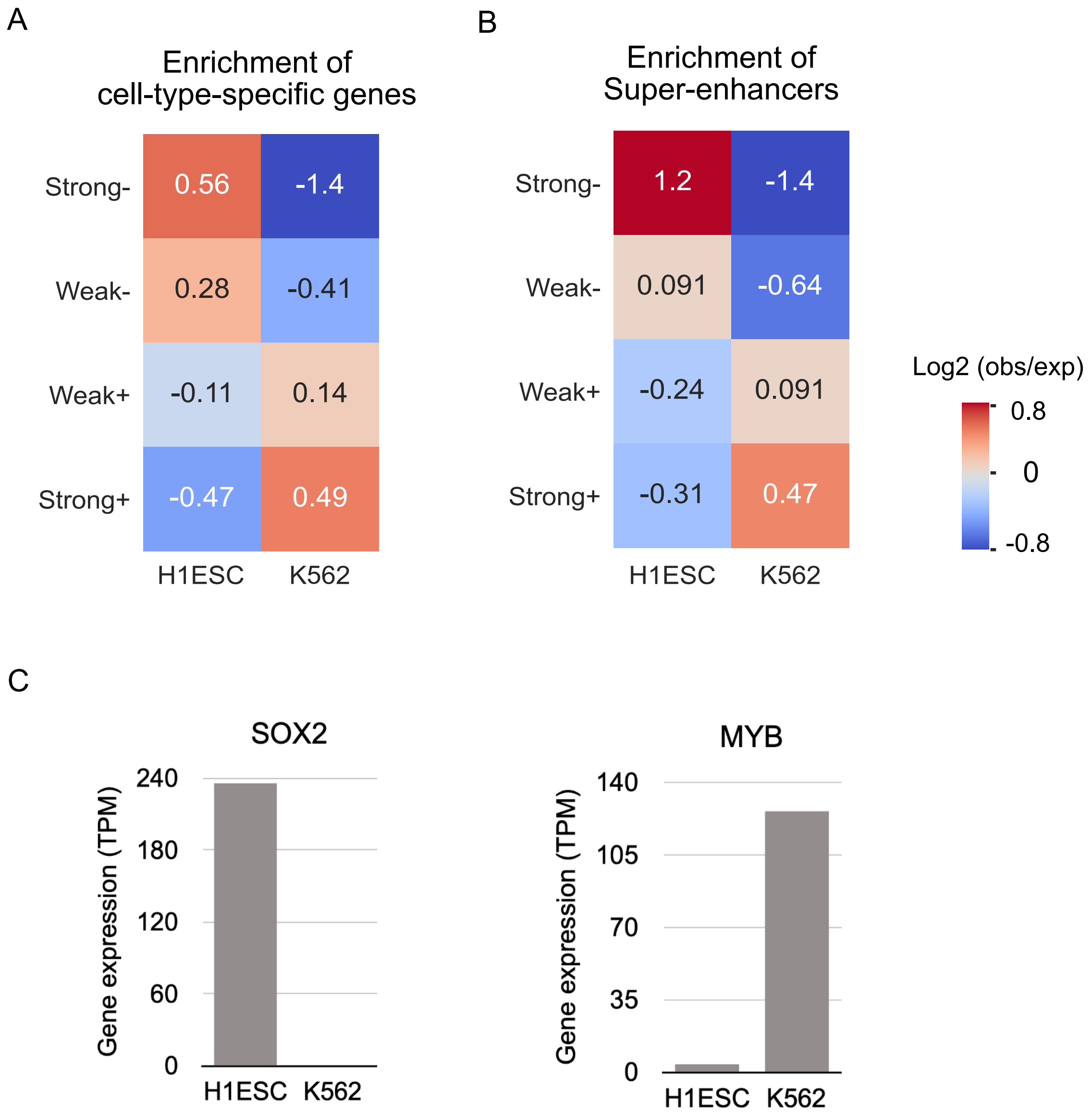

Supplementary Figure 3

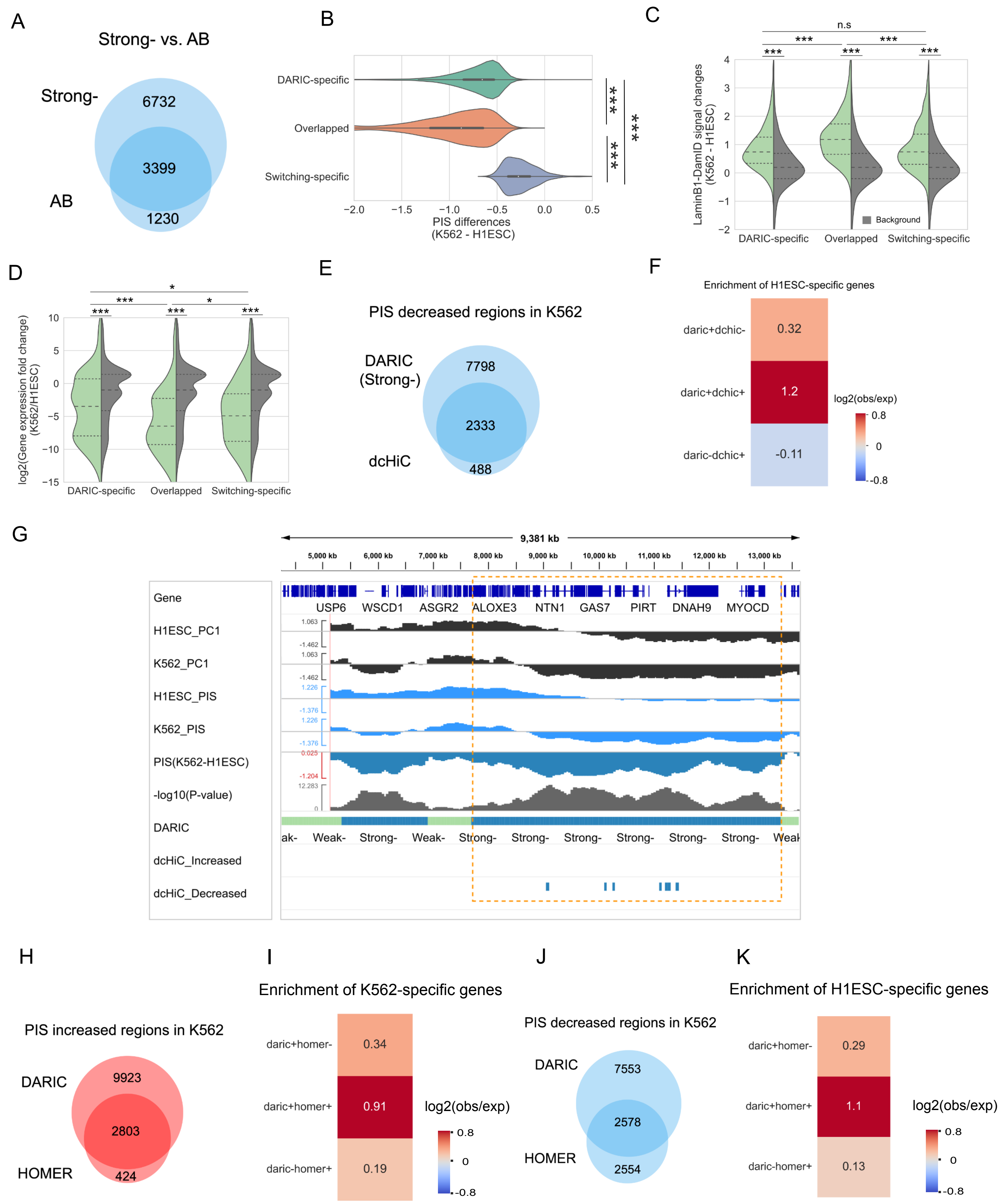

Supplementary Figure 4

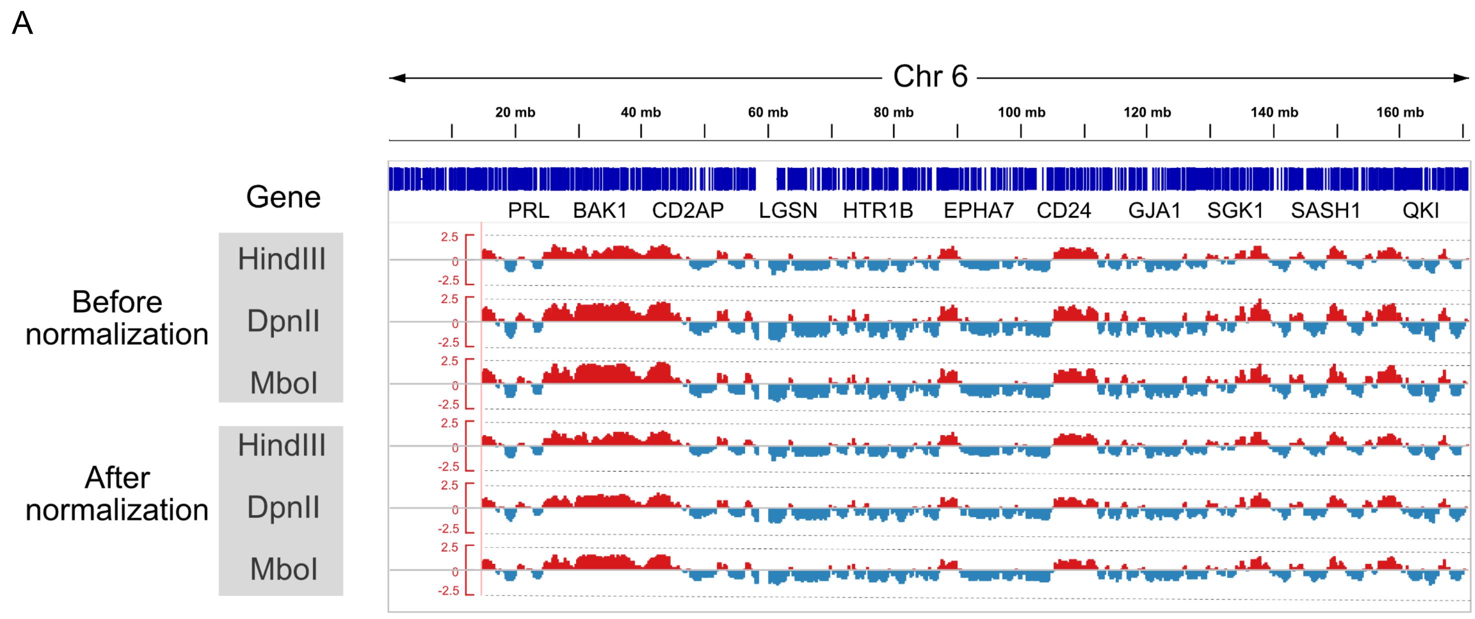

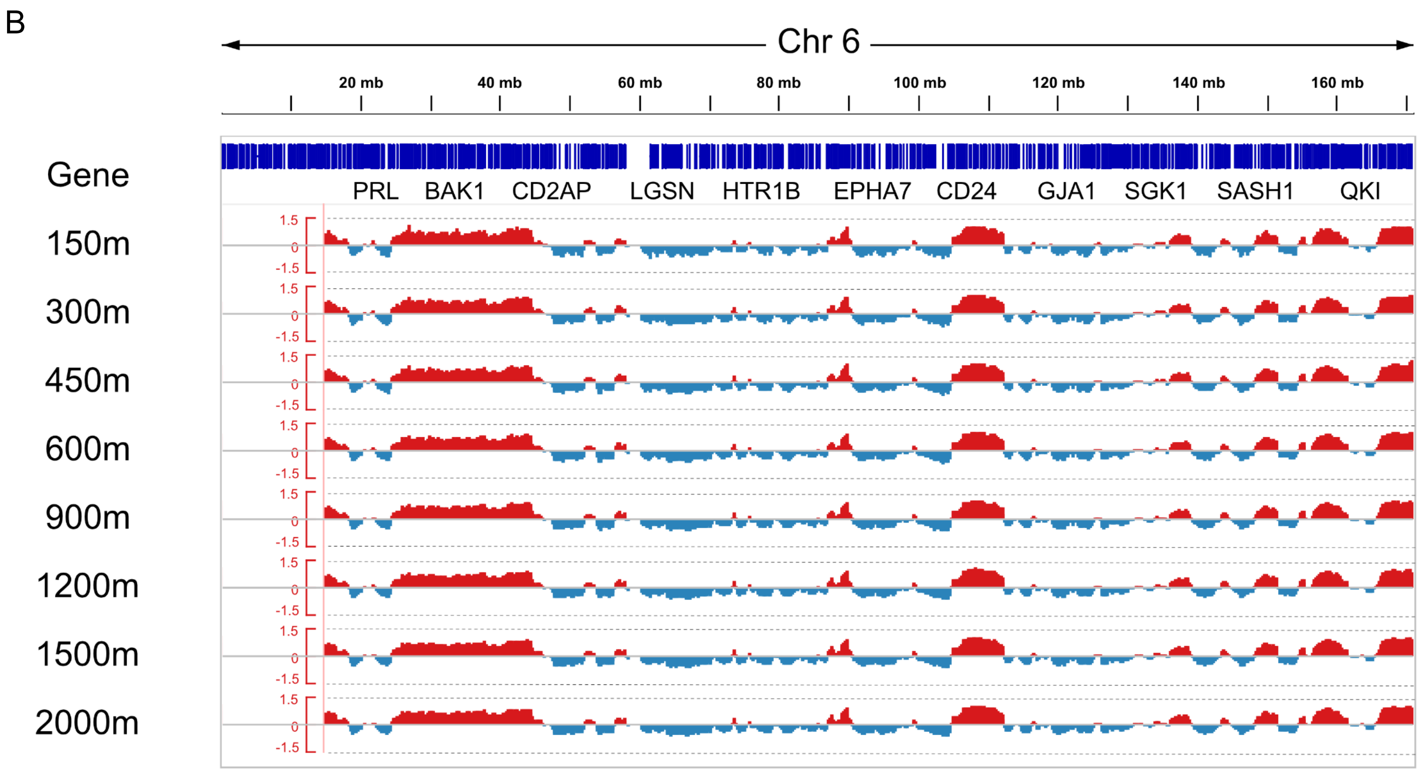

Supplementary Figure 5

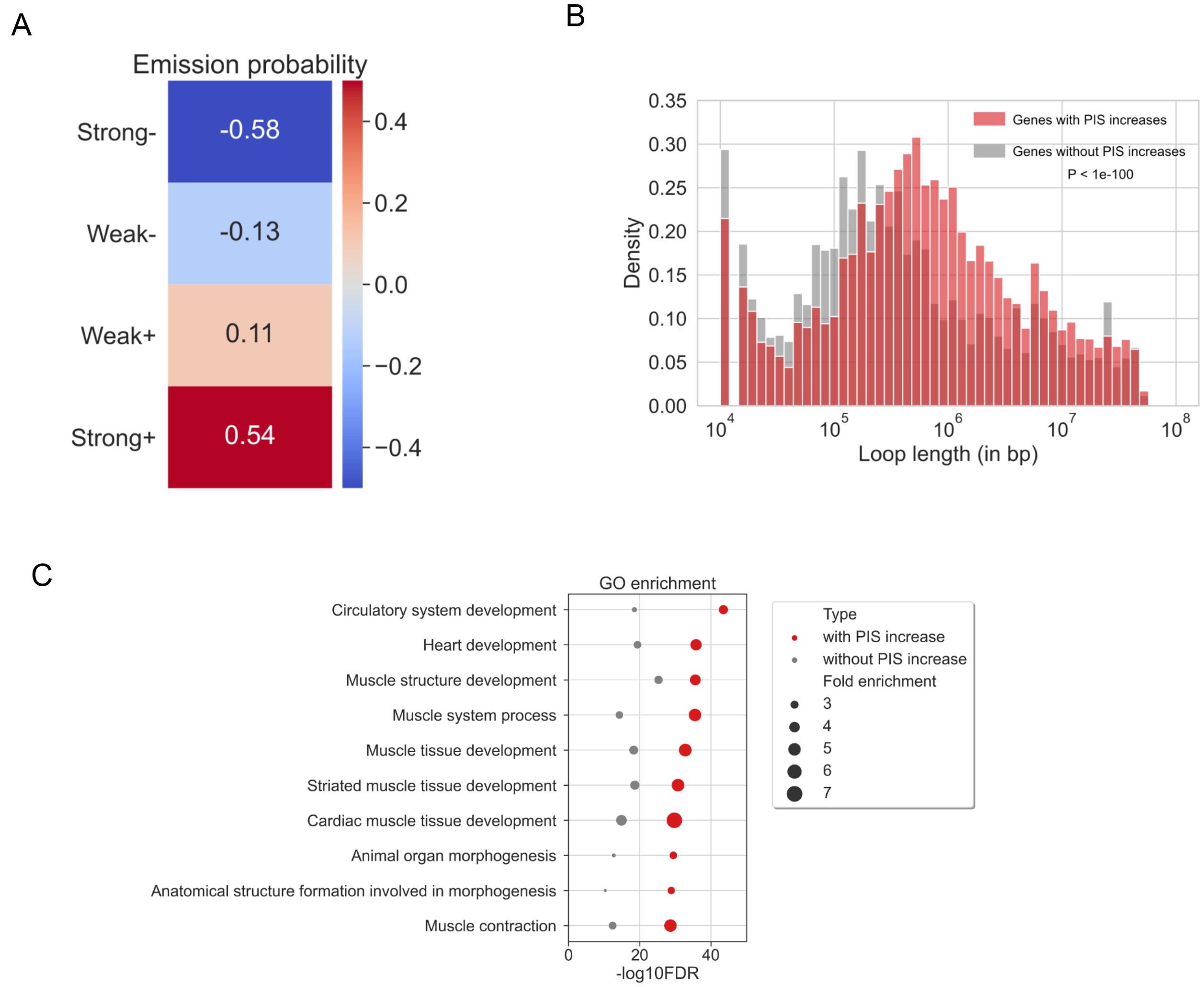

Supplementary Figure 6

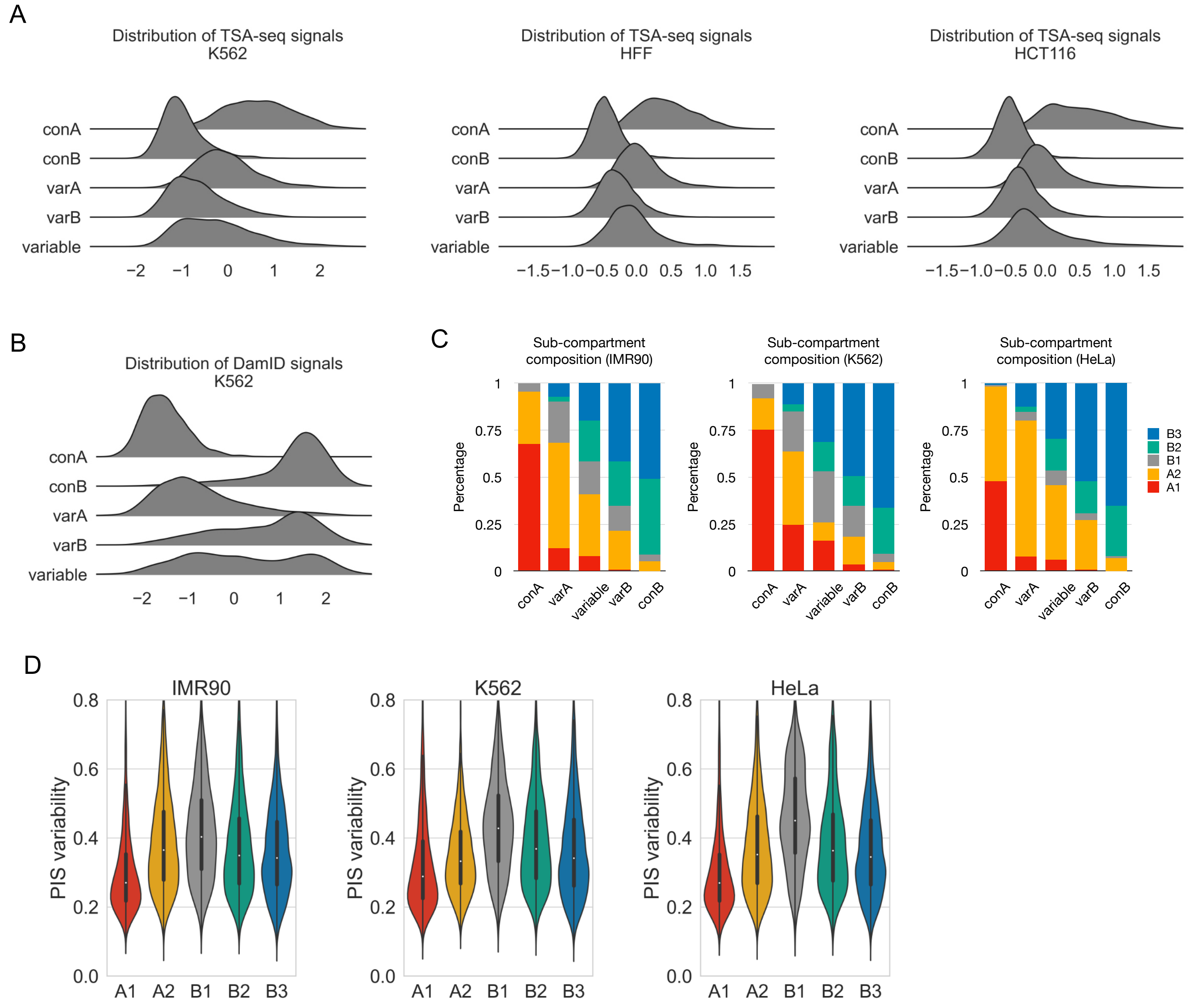

Supplement: Supplementary file 1 — Additional file 1: Figure 1. Introduction of the DARIC framework. A Scatter plot showing the high correlation between PIS and PC1 values from the H1ESC Hi-C data. B MA plot showing the systematic differences between H1ESC and K562 cells. Each dot represents a 50kb bin. The Red dashed line represents the fitted line from the M and A values. C MA plot after normalization showing the elimination of the systematic differences between the two cell types. D-E The emission matrix (D) and state coverage matrix (E) for the 5-state HMM model. F Confusion matrix showing the overlap between the states of 5-state model and those of the 4-state model. Numbers represent 50kb bins. Figure 2. Functional association between gene regulation and differential compartments revealed by DARIC. A-B Heatmap showing the enrichment of cell type-specific genes (A) and superenhancers. (B) in the four states identified by DARIC. Values show the log2(observed/expected) enrichment. C Bar plots showing the expression of SOX2 and MYB genes in H1ESC and K562 cells. Figure 3. Comparison between DARIC and existing methods. A Venn diagram presenting the overlap between the ‘Strong-’ state revealed by DARIC and the ‘AB’ state in conventional analyses. The numbers in the plot represent the numbers of 50kb bins. B Violin plot showing the PIS differences for the three types of domains defined in (A). C-D Violin plots showing the comparisons of Lamina1-DamID signal changes (C), and gene expression fold changes (D) in the three types of domains defined in (A). E Venn diagram showing the overlap of genomic bins identified with decreased PIS/PC1 values in K562 by DARIC and dcHiC. Numbers of 50kb bins were shown in the diagram. F Enrichment of H1ESC-specific genes for the three types of genomic regions defined in (E). G An exemplary region showing DARIC and dcHiC output with decreased PIS in K562 cells. H-K Performance comparison between DARIC and HOMER using H1ESC versus K562 as an example. (H) Venn diagram show [file 12864_2023_9675_MOESM1_ESM.pdf]
