# Supplementary material for: Identifying quantitatively differential chromosomal compartmentalization changes and their biological significance from Hi-C data using DARIC
Source: BMC Genomics. 2023 Oct 13;24:614. doi: 10.1186/s12864-023-09675-w (PMC10571287; doi:10.1186/s12864-023-09675-w)
Supplement: Supplementary file 2 — Additional file 2: Table S1. Data used for the H1ESC-K562 comparison. [file 12864_2023_9675_MOESM2_ESM.docx]

**Supplementary Table 1**. Data used for the H1ESC-K562 comparison

| Data type | Replicate | Cell | Accession | Lab | Source study |
| --- | --- | --- | --- | --- | --- |
| Hi-C | Rep1 | H1ESC | 4DNFIINYRY8H | Job Dekker | PMID:32213324 |
|  | Rep2 | H1ESC | 4DNFILR6CKEJ | Job Dekker | PMID:32213324 |
|  | Rep1 | K562 | 4DNFI4DGNY7J  4DNFI9G9FRJJ | Erez Lieberman Aiden | PMID:25497547 |
|  | Rep2 | K562 | 4DNFI7CIF2JL  4DNFID8N3SCC  4DNFIKFV5Y88  4DNFIRHH2E7D | Erez Lieberman Aiden | PMID:25497547 |
| RNA-seq | Rep1 | H1ESC | ENCFF619SCD | Barbara Wold | PMID: 32728249 |
|  | Rep2 | H1ESC | ENCFF951UFS | Barbara Wold | PMID: 32728249 |
|  | Rep1 | K562 | ENCFF671NWM | Barbara Wold | PMID: 32728249 |
|  | Rep2 | K562 | ENCFF492ENI | Barbara Wold | PMID: 32728249 |
| DNase-seq | Rep1 | H1ESC | ENCFF915WAL | Gregory Crawford | PMID: 32728249 |
|  | Rep1 | K562 | ENCFF972GVB | Gregory Crawford | PMID: 32728249 |
| H3K27ac ChIP-seq | Rep1 | H1ESC | ENCFF860ABR | Bradley Bernstein | PMID: 32728249 |
|  | Rep1 | K562 | ENCFF121RHF | Bradley Bernstein | PMID: 32728249 |
| TSA-seq | Rep1 | H1ESC | 4DNFI625PP2A | [Andrew Belmont](https://data.4dnucleome.org/labs/andrew-belmont-lab/) | [PMID:33355299](https://data.4dnucleome.org/publications/b3bf2d54-4ac9-4808-b44d-82c35996052f/) |
|  | Rep1 | K562 | 4DNFIVZSO9RI | [Andrew Belmont](https://data.4dnucleome.org/labs/andrew-belmont-lab/) | [PMID:33355299](https://data.4dnucleome.org/publications/b3bf2d54-4ac9-4808-b44d-82c35996052f/) |
| Lamin B1 DamID | Rep1 | H1ESC | 4DNFIXNBG8L1 | [Bas van Steensel](https://data.4dnucleome.org/labs/bas-van-steensel-lab/) | PMID: 28905911 |
|  | Rep1 | K562 | 4DNFIX4BXSIM | [Bas van Steensel](https://data.4dnucleome.org/labs/bas-van-steensel-lab/) | PMID: 28905911 |
